# Nuclear PTEN functions as an essential regulator of SRF-dependent transcription to control smooth muscle differentiation

**DOI:** 10.1038/ncomms10830

**Published:** 2016-03-04

**Authors:** Henrick Horita, Christina L. Wysoczynski, Lori A. Walker, Karen S. Moulton, Marcella Li, Allison Ostriker, Rebecca Tucker, Timothy A. McKinsey, Mair E. A. Churchill, Raphael A. Nemenoff, Mary C. M. Weiser-Evans

**Affiliations:** 1 Department of Medicine, Division of Renal Diseases and Hypertension, University of Colorado, Anschutz Medical Campus, 12700 East 19th Avenue, C281, Research Complex 2, Room 7101, Aurora, Colorado 80045, USA; 2 Department of Pharmacology, University of Colorado, Anschutz Medical Campus, Aurora, Colorado 80045, USA; 3 Department of Medicine, Division of Cardiology, University of Colorado, Anschutz Medical Campus, Aurora, Colorado 80045, USA; 4 Center for Fibrosis Research and Translation, School of Medicine, University of Colorado, Anschutz Medical Campus, Aurora, Colorado 80045, USA; 5 Department of Medicine, Cardiovascular Pulmonary Research Program, University of Colorado, Anschutz Medical Campus, Aurora, Colorado 80045, USA

## Abstract

Vascular disease progression is associated with marked changes in vascular smooth muscle cell (SMC) phenotype and function. SMC contractile gene expression and, thus differentiation, is under direct transcriptional control by the transcription factor, serum response factor (SRF); however, the mechanisms dynamically regulating SMC phenotype are not fully defined. Here we report that the lipid and protein phosphatase, PTEN, has a novel role in the nucleus by functioning as an indispensible regulator with SRF to maintain the differentiated SM phenotype. PTEN interacts with the N-terminal domain of SRF and PTEN–SRF interaction promotes SRF binding to essential promoter elements in SM-specific genes. Factors inducing phenotypic switching promote loss of nuclear PTEN through nucleo-cytoplasmic translocation resulting in reduced myogenically active SRF, but enhanced SRF activity on target genes involved in proliferation. Overall decreased expression of PTEN was observed in intimal SMCs of human atherosclerotic lesions underlying the potential clinical importance of these findings.

## Abstract (web summary)

The transcription factor, serum response factor, SRF regulates critical smooth muscle (SM) contractile gene expression but what else controls SM differentiation is unclear. Here, Horita *et al*. demonstrate that nuclear PTEN acts with SRF at the transcriptional level to maintain the differentiated SM phenotype.

## Graphical abstract

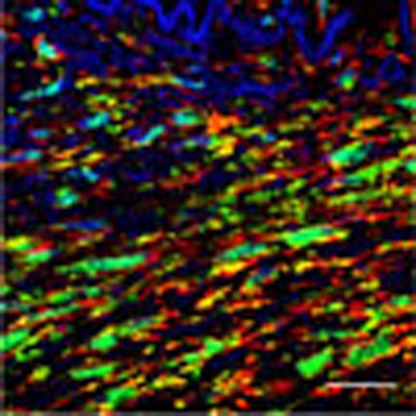

Cardiovascular diseases are the leading causes of death in industrialized nations. Atherosclerosis is a chronic inflammatory disease that progresses to complex, unstable arterial lesions^1^,^2^. Restenosis is an acute inflammatory vascular disease and a major limitation of percutaneous angioplasty procedures, especially in higher risk patient populations^3^,^4^. Both are characterized by activation of vascular smooth muscle cells (SMCs) resulting in an inflammatory environment, neointimal hyperplasia and vessel occlusion^1^,^2^,^3^,^4^,^5^. Under physiologic conditions, SMCs express a quiescent, differentiated phenotype distinguished by high levels of SMC-specific contractile proteins (for example, SM-alpha-actin (*Acta2*/αSMA) and SM myosin heavy chain (*Myh11*/SM-MHC))^6^,^7^. SMC activation promotes a transition to a highly proliferative, inflammatory phenotype characterized by downregulation of SM genes and increased production of multiple cytokines and chemokines (that is, SMC dedifferentiation)^5^,^6^,^8^,^9^,^10^,^11^,^12^,^13^. Collectively, existing evidence supports the concept that resident SMCs serve as both initiators and effectors thereby playing a multifaceted role in the progression of vascular disease.

SMC differentiation is associated with serum response factor (SRF)-dependent transcriptional activation of SM contractile genes, thereby conferring the distinctive physiological characteristics of SMCs. SRF is a transcription factor that binds CArG box elements in promoter regions of target genes^14^,^15^,^16^. In SMCs, SRF regulates two distinct gene programs, SM contractile genes and growth-related immediate early genes (IEG; for example, *Fos*/c-fos)^14^. While seemingly paradoxical, identification of specific SRF co-factors that promote SM contractile (myocardin) or SMC proliferative (ETS-like transcription factor 1 (Elk-1)) programs resolved the contradiction^17^,^18^,^19^,^20^,^21^,^22^,^23^,^24^. Yet questions remain regarding the mechanisms and factors responsible for maintaining the SMC differentiation program particularly *in vivo*. Recent findings also demonstrated that the ability of SRF and co-factors to engage essential CArG sites of SM target genes requires epigenetic modifications of chromatin^5^,^25^,^26^,^27^,^28^. Cellular spatial regulation may be another potential mechanism controlling SRF activity. SRF nuclear exclusion (NES) resulted in decreased SM gene expression in airway SMC^29^ and we showed that PDGF represses SRF activity and translocates SRF out of the nucleus in vascular SMC^30^. SRF has a higher affinity for IEG promoters compared with SM gene promoters, likely due to degenerate CArG elements in SM gene promoters; thus, where SRF levels are a limiting factor, this may be a critical determinant for gene program activation. Despite the work in the field, however, a clear mechanistic understanding of SMC phenotype control, how phenotypic switching is dynamically regulated and mechanisms mediating cell specificity is lacking.

The concept of SMC phenotypic modulation is well-accepted and plays an essential role in vascular disease progression. The mechanisms regulating SM gene repression are complex yet a complete understanding, while a challenge, is critical to enable therapeutic advances in the treatment of vascular diseases. Although multiple stimuli dedifferentiate SMCs, the underlying molecular programs actively repressing dedifferentiation remain unclear. PTEN is a dual-specificity protein and lipid phosphatase that suppresses numerous signalling networks is involved in cell proliferation, survival and inflammation. We and others showed that PTEN inactivation promotes an activated SMC phenotype characterized by increased proliferation, increased inflammatory cytokine production, decreased SM gene expression and vascular disease progression^31^,^32^,^33^,^34^,^35^,^36^,^37^,^38^. PTEN classically functions as a cytoplasmic lipid phosphatase to antagonize PI3-kinase/Akt-mediated signalling^39^,^40^,^41^,^42^,^43^,^44^. Our previous work demonstrated that effects on SMC proliferation and cytokine production are phosphatase-dependent and mediated by Akt-induced NFκB and HIF-1α activity^35^,^36^,^38^. In addition, we showed that PTEN is a downstream effector of SRF through a microRNA (miRNA)-dependent pathway. Loss of an SRF–PTEN axis promotes reprogramming of SMCs into a proliferative, inflammatory phenotype^37^. However, inhibition of Akt signalling only partially restored SM gene expression in the setting of PTEN depletion^36^. Since SM gene expression is under direct transcriptional control by SRF, the mechanism underlying PTEN's regulation of the SMC differentiation program remained unclear. Emerging data in other cell systems support a role for nuclear PTEN both in a phosphatase-dependent and -independent manner thereby uncovering a function for PTEN independent of its cytoplasmic Akt-antagonizing effects^45^,^46^,^47^,^48^,^49^,^50^,^51^,^52^,^53^. However, there is no information regarding the biological significance of nuclear PTEN in SMCs.

We describe here a novel and unanticipated function for PTEN in transcriptional control through association with SRF and its muscle-specific co-factor, myocardin. This association facilitates selective binding of SRF on the *Myh11* and *Acta2* promoters, but not the *Fos* promoter, thus activating SMC contractile gene expression. We used mouse genetic models and *in vitro* approaches to demonstrate that PTEN is an indispensable regulator of SRF that plays a key role in SRF transcriptional activity as a mechanism to dynamically regulate SM contractile genes. In addition, we report overall decreased expression of PTEN in intimal SMCs of a small cohort of human atherosclerotic lesions underlying the potential clinical significance of our findings.

## Results

### Aortic contractility is reduced in PTEN iKO mice

**Figure 1:**
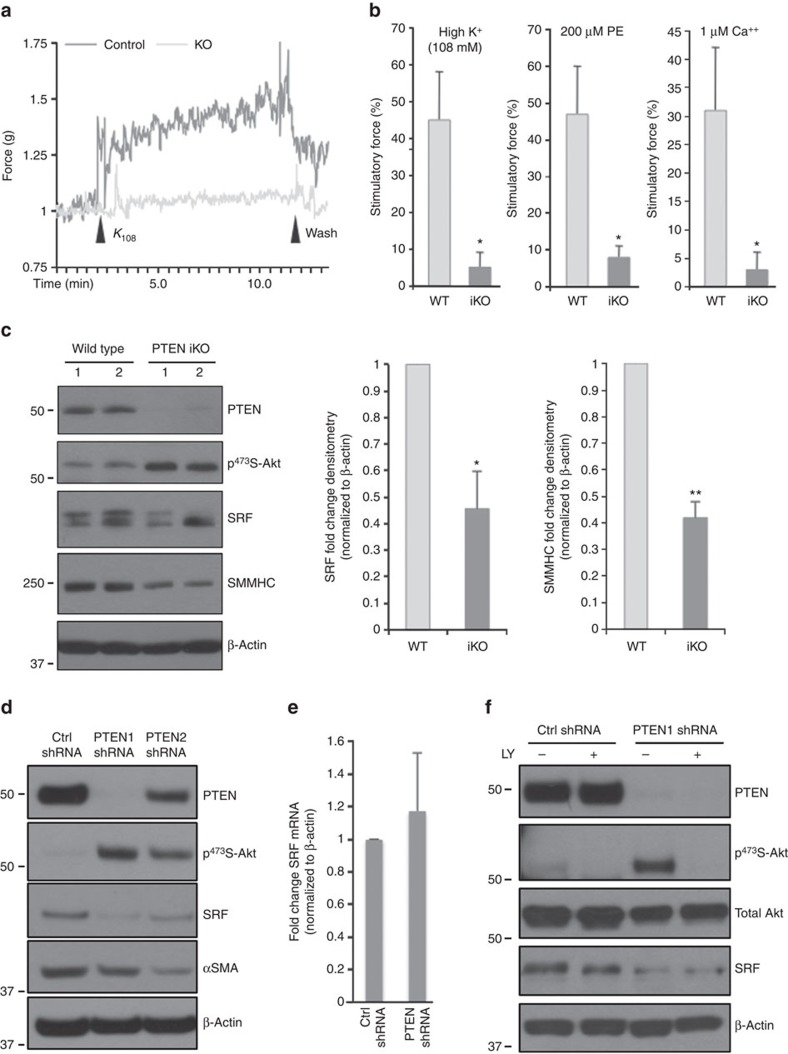
PTEN-dependent vessel contractility and serum response factor (SRF) activity. Wild-type (WT) and inducible PTEN knockout (PTEN iKO) mice were generated and treated with tamoxifen as described in Methods. (**a**,**b**) Isometric force normalized to vessel length was measured in isolated aortic rings from WT and PTEN iKO mice exposed to the indicated concentrations of potassium chloride (K^+^), phenylephrine (PE) or calcium chloride (Ca^++^). (**a**) Representative tracing from potassium-stimulated aortic rings. (**b**) Quantification of force generation. Data represent averages±s.e.m. from six (K^+^, PE) or four (Ca^++^) vessels per group. *P<0.01 versus WT. (**c**) Western blot analysis for PTEN, phospho-Akt, SRF and SM myosin heavy chain (SM-MHC) in whole cell lysates (WCL) of aortic media from WT or PTEN iKO mice. β-Actin was used as a loading control. Left—representative blot from two mice per genotype; each lane represents an individual mouse. Right—fold changes in densitometry measurements±s.e.m. *N*=12; *P=0.016; **P=0.000056 versus WT. (**d**,**e**) Smooth muscle cells (SMCs) stably expressing control (Ctrl) or PTEN-specific shRNA were serum-restricted for 24 h (RNA) or 48 h (protein). (**d**) WCL were analysed for total PTEN, phospho-Akt, SRF and αSMA. (**e**) Total RNA was analysed by qPCR for SRF mRNA. Shown are fold changes in SRF mRNA copy number±s.e.m. from six independent experiments. (**f**) Ctrl and PTEN-deficient SMCs were serum-restricted in the presence or absence of the PI3-kinase inhibitor, LY294002 (10 μM) for 24 h. WCL were analysed for total PTEN, phospho-Akt, total Akt and SRF levels. *N*=3 independent experiments. Molecular weight markers were cropped out for final SRF blots; please see Supplementary Fig. 8. qPCR, quantitative PCR.

Abnormal contractility occurs in atherosclerotic and restenotic vessels. We showed that PTEN deficiency *in vitro* promotes a dedifferentiated phenotype characterized molecularly by decreased expression of SM contractile genes^36^. Here to determine if normal vessel contractility is impaired by PTEN deficiency, we used an inducible SMC-specific PTEN knockout (PTEN iKO) mouse model previously generated in our lab^38^. Vascular reactivity of intact aortic rings isolated from wild-type (WT) or PTEN iKO mice was measured as described in Methods. Aortic rings from PTEN iKO mice exhibited 9- and 5.8-fold decreases in maximal contractile force in response to KCl and phenylephrine (PE), respectively, compared with WT mice (Fig. 1a,b). Maximal contractile force in response to Ca^++^ was decreased 10-fold in aortic rings from PTEN iKO mice compared with WT mice (Fig. 1b), suggesting an intrinsic impairment of the contractile machinery by PTEN deficiency. Decreased vessel contractility was associated with decreased expression of SM-MHC in aortic media from PTEN iKO mice compared with WT mice (Fig. 1c), in agreement with our published *in vitro* data^36^. Surprisingly, decreased SRF expression was also observed (Fig. 1c).

### PTEN regulates SRF protein levels and transcriptional activity

To determine the mechanism underlying PTEN's effect on SM gene expression, PTEN-specific short hairpin RNA (shRNA) was used to selectively reduce PTEN in cultured SMCs. We observed reduced levels of SRF protein in PTEN-deficient SMCs compared with controls (Fig. 1d), in agreement with the *in vivo* data. There were no changes in SRF mRNA indicating SRF was not regulated at the level of transcription (Fig. 1e). Treatment of PTEN-deficient SMCs with the PI3K inhibitor, LY294002, had no effect on SRF levels (Fig. 1f) indicating a lipid phosphatase-independent effect of PTEN on SRF expression. In contrast, inhibition of proteasomal degradation restored SRF protein levels in PTEN-deficient SMCs, suggesting PTEN loss results in proteasome-mediated SRF degradation (Supplementary Fig. 1a). To determine if PTEN overexpression is sufficient to enhance SRF transcriptional activity, SMCs were transduced with adenoviruses expressing empty vector (EV), WT PTEN or lipid/protein phosphatase-inactive PTEN (MT). Compared with EV, overexpression of both WT and MT PTEN increased SRF protein and, importantly, increased expression of the SRF target gene, αSMA (Supplementary Fig. 1b) supporting a role for PTEN in regulation of SRF transcriptional activity. In contrast, overexpression of PTEN in HEK 293 cells or L929 fibroblasts had no effect on SRF protein levels or SM gene induction (Supplementary Fig. 2a–c) suggesting a selective effect of PTEN in SMCs. As inhibition of PTEN-regulatable Akt activity did not restore SRF in PTEN-deficient SMCs and both WT and MT PTEN increased SRF transcriptional activity, our data suggest a novel role for PTEN independent of its known lipid or protein phosphatase activity.

### PTEN, SRF, and myocardin form a multi-protein complex

**Figure 2:**
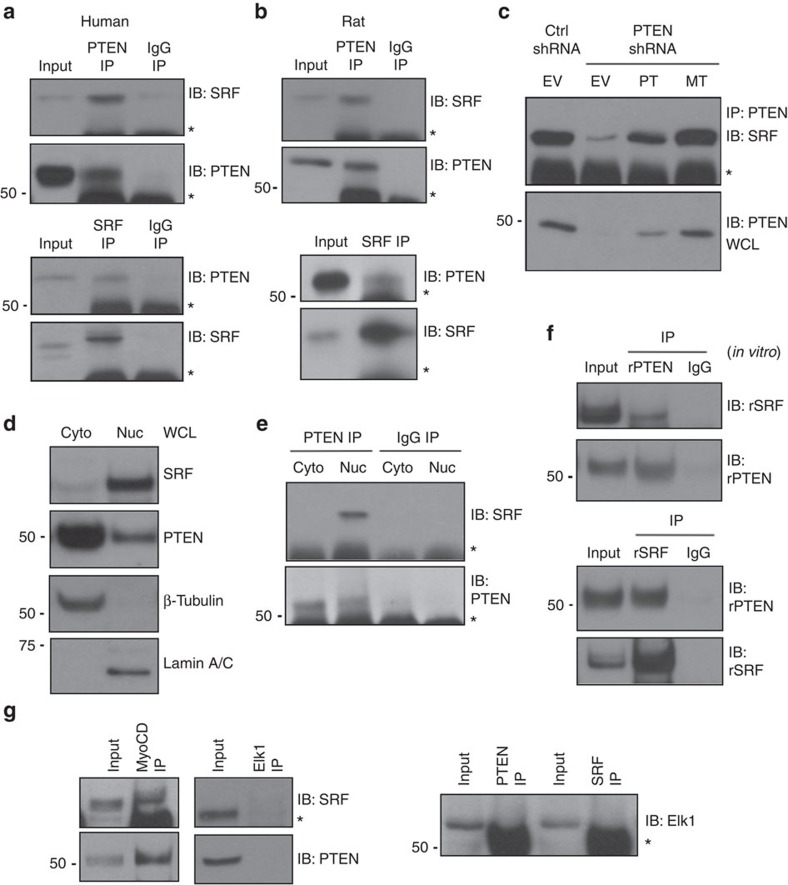
PTEN forms a nuclear multi-protein complex with SRF and myocardin. (**a**,**b**) PTEN (top) or SRF (bottom) proteins were immunoprecipitated (IP) from WCL of cultured human (**a**) or rat (**b**) aortic SMCs serum restricted for 48 h. About 10% of input WCL and co-immunoprecipitating SRF or PTEN were detected by immunoblotting (IB). A non-specific IgG was used for IPs as a negative control. (**c**) SMCs stably expressing control (Ctrl) or PTEN-specific shRNA were transiently transduced with empty vector adenovirus (EV) or adenoviruses encoding wild-type PTEN (WT) or phosphatase-inactive PTEN (MT) (multiplicity of infection=100). PTEN was immunoprecipitated (IP) and co-immunoprecipitating SRF was detected by immunoblotting (IB). (**d**) Cytoplasmic (cyto) and nuclear (nuc) SMC extracts were analysed by western blotting for SRF and PTEN levels. β-Tubulin and Lamin A/C were used as cytoplasmic and nuclear loading controls, respectively. (**e**) PTEN was immunoprecipitated from cytoplasmic and nuclear SMC extracts and co-immunoprecipitating SRF was detected by immunoblotting. A non-specific IgG was used for IPs as a negative control. (**f**) Recombinant His-tagged PTEN and GST-tagged SRF were purified, incubated together, and PTEN (top) or SRF (bottom) were immunoprecipitated. Co-immunoprecipitating SRF or PTEN was detected by immunoblotting. About 10% of protein mixture was immunoblotted to control for input. A non-specific IgG was used for IPs as a negative control. (**g**) Myocardin (left), Elk-1 (middle) and SRF or PTEN (right) proteins were immunoprecipitated from WCL of SMCs serum restricted for 48 h. About 10% of input WCL and co-immunoprecipitating SRF, PTEN or Elk-1 were detected by immunoblotting. Shown are representative blots from a minimum of three independent experiments. *, heavy chain IgG. Molecular weight markers were cropped out for final SRF blots; please see Supplementary Fig. 8.

The lipid phosphatase, PI3K–Akt-antagonizing activity of PTEN occurs in the cytoplasm. Recent data, however, support important biological roles for nuclear PTEN through direct interactions with nuclear factors and independent of its phosphatase activity^50^ or as a nuclear protein phosphatase that directly targets transcription factors to activate them^46^,^51^. To determine if PTEN regulates SRF through interaction between the proteins, reciprocal co-immunoprecipitation (co-IP) assays were performed. We found PTEN immunoprecipitated SRF and, similarly, SRF immunoprecipitated PTEN in both human- and rat-derived aortic SMCs (Fig. 2a,b); complex formation was specific as no interaction was observed with the use of a non-specific IgG. Protein–protein binding was independent of PTEN's phosphatase activity as rescue of PTEN levels in PTEN-depleted SMCs using either WT or MT PTEN restored PTEN–SRF interactions (Fig. 2c). Cell fractionation showed that PTEN localizes to both the cytoplasm and nucleus in SMCs, with the majority present in the cytoplasm (Fig. 2d). However, under basal conditions PTEN interaction with SRF occurred predominantly in the nucleus (Fig. 2e). *In vitro* binding assays using bacterially expressed and purified amino terminal-tagged His–PTEN and glutathione-S-transferase (GST)–SRF were conducted to determine whether this interaction is direct (Supplementary Fig. 3). Compared with a non-specific IgG negative control, reciprocal co-IPs demonstrated that in *in vitro* conditions PTEN directly interacted with SRF (Fig. 2f). SRF controls SM gene or IEG expression through selective interactions with the muscle-restricted cofactor, myocardin or the ETS-domain family member, Elk-1, respectively. Co-IPs were performed to determine if PTEN forms a higher order protein complex selectively with myocardin to regulate SRF-dependent SM gene expression. Consistent with SMCs expressing a differentiated phenotype, interactions between myocardin and SRF and myocardin and PTEN were readily detected, but not between Elk-1 and SRF or Elk-1 and PTEN (Fig. 2g).

The identification of myocardin significantly advanced our understanding of how SM gene transcription is controlled by SRF, a widely expressed transcription factor. However, myocardin, exclusively expressed in cardiac and smooth muscle, is essential for both cardiac and SM contractile gene transcription^20^,^54^. To determine if multi-protein complex formation was specific to SMCs or more general to muscle gene expression, neonatal rat ventricular cardiomyocytes (NRVMs) were cultured under basal conditions or in response to hypertrophic stimuli (PE). As expected, both PTEN and SRF were readily detectable in NRVMs under both conditions; compared with basal, no differences in expression were observed in response to PE (Supplementary Fig. 4a). In contrast to SMCs, PTEN and SRF did not interact in NRVMs under basal conditions, although PE promoted a strong interaction between PTEN and SRF similar to that observed in SMCs (Supplementary Fig. 4b) suggesting a role for PTEN in agonist-mediated cardiomyocyte hypertrophy. While PTEN overexpression in L929 fibroblasts was not sufficient to promote SM gene induction (Supplementary Fig. 2c), similar to SMCs and PE-stimulated NRVMs, SRF immunoprecipitated with PTEN in these cells indicating an uncoupling of PTEN-dependent SRF-mediated SM gene transcription in non-muscle cells (Supplementary Fig. 4c).

### PTEN is essential for SRF binding on SM gene promoters

**Figure 3:**
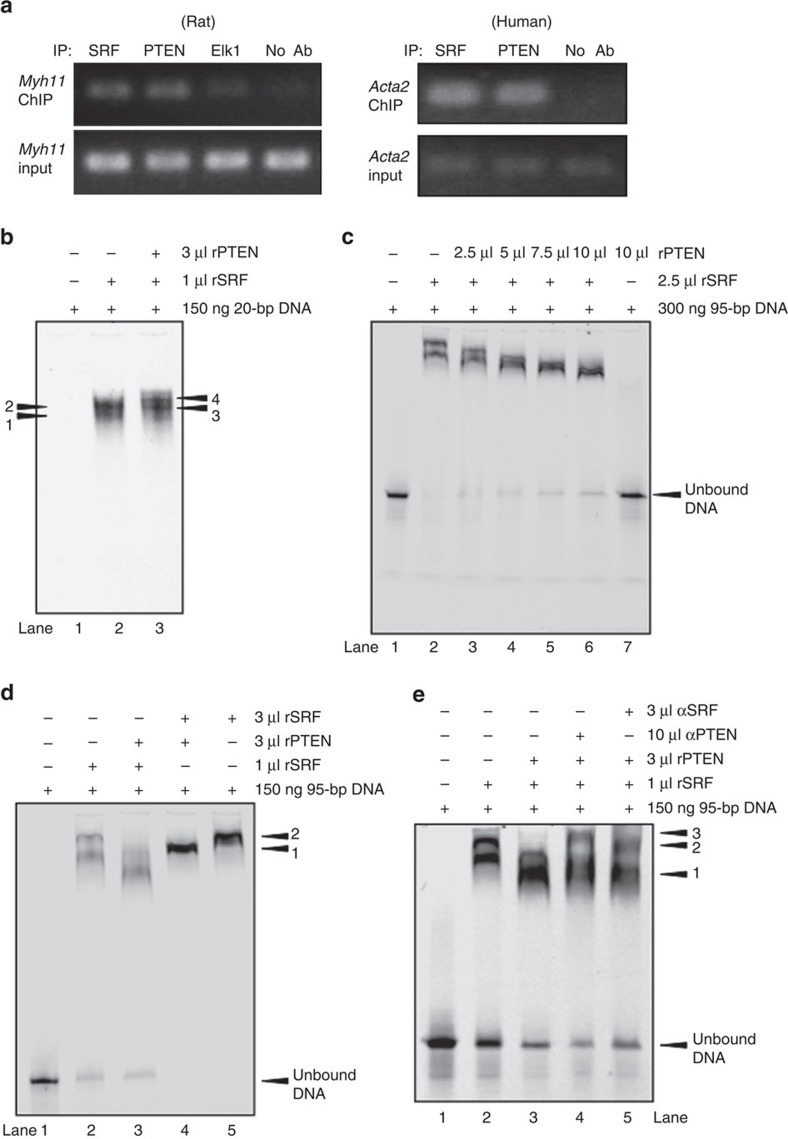
PTEN–SRF interact with CArG boxes of SM genes. (**a**) Chromatin immunoprecipitation (ChIP) analyses for protein binding to the *Myh11* or *Acta2* promoters. DNA from serum-restricted rat (left) or human (right) SMCs was cross-linked with formaldehyde and recovered from immunoprecipitated samples using SRF, PTEN or Elk-1 (rat SMCs only) antibodies or a no-antibody negative control. Immunoprecipitated DNA was subjected to qPCR amplification using primers flanking essential CArG boxes in the *Myh11* and *Acta2* promoters. About 2% genomic DNA input was used as a positive control. (**b**–**e**) Electrophoretic mobility shift assays (EMSAs) were conducted as described in Methods using the indicated amount of a fluorescently labelled 20-bp DNA fragment containing CArG ‘B' of the *Acta2* promoter (b) or a 95-bp DNA fragment consisting of CArGs ‘A' and ‘B' of the *Acta2* promoter (**c**–**e**) and the indicated volumes of purified recombinant SRF and PTEN. (**b**) EMSA with 20-bp DNA fragment. Positions of SRF-containing complexes are labelled 1 and 2 (lane 2); positions of SRF–PTEN-containing complexes are labelled 3 and 4 (lane 3); unbound DNA not shown. (**c**) EMSA with 95-bp DNA fragment. DNA plus rSRF alone (lane 2) compared with DNA plus rSRF and increasing amounts of rPTEN (lanes 3–6). Lane 7 shows DNA plus rPTEN alone. (**d**) EMSA with 95-bp DNA fragment. DNA plus 1 μl rSRF (lane 2) or saturating amounts (3 μl) of rSRF (lane 5) compared with DNA plus 3 μl rPTEN and 1 μl rSRF (lane 3) or saturating amounts (3 μl) of rSRF (lane 4). Position of SRF-containing complexes are labelled ‘2' (lane 5); position of SRF–PTEN-containing complexes are labelled ‘1' (lane 4). (**e**) EMSA with 95-bp DNA fragment. DNA plus 1 μl rSRF (lane 2), DNA plus 3 μl rPTEN and 1 μl rSRF (lane 3), DNA plus 3 μl rPTEN and 1 μl rSRF (lane 3) supershifted with a PTEN-specific antibody (lane 4), and DNA plus 3 μl rPTEN and 1 μl rSRF (lane 3) supershifted with an SRF-specific antibody (lane 5). Position of SRF–PTEN-containing complexes are labelled ‘1', position of SRF–PTEN-containing complexes supershifted with a PTEN antibody are labelled ‘3' and position of SRF–PTEN-containing complexes supershifted with an SRF antibody are labelled ‘2'. Shown for each panel are representative images from a minimum of three independent experiments. qPCR, quantitative PCR.

In addition to co-factor interaction, SM gene transcription involves direct association of SRF and co-factors with CArG box chromatin of SM genes. To determine if PTEN facilitates SRF binding, chromatin immunoprecipitation (ChIP) combined with quantitative PCR was conducted. Compared with no-antibody control, binding of both SRF and PTEN to essential CArG boxes in the *Myh11* and *Acta2* promoters of WT rat-derived (*Myh11*) and human-derived (*Acta2*) SMCs under basal conditions was detected (Fig. 3a). In contrast, binding of PTEN to CArG elements in the *Fos* promoter was not detected (not shown). To strengthen the evidence that PTEN forms a higher order SRF–CArG complex, electrophoretic mobility shift assays (EMSAs) were conducted using purified recombinant PTEN (rPTEN) and recombinant SRF (rSRF) and a 20-bp probe representing CArG ‘B' or a 95-bp DNA probe consisting of CArG ‘A' and CArG ‘B' elements of the *Acta2* promoter, as described previously^55^. As expected using the single CArG-containing 20-bp probe, an SRF-containing DNA-binding complex was formed with addition of rSRF alone (Fig. 3b). Importantly, a lower mobility protein–DNA-binding complex formed when rPTEN was added (Fig. 3b). Using the multi-CArG-containing 95-bp probe, two bands formed with the addition of rSRF alone (Fig. 3c, lane 2). Titration of increasing amounts of rSRF resulted in the formation of a single high mobility band at low SRF levels, two lower mobility bands with increased SRF levels and a single low mobility band at the highest levels of SRF, presumably due to increased occupancy of CArG elements by SRF (Supplementary Fig. 5a). Surprisingly, using the multi-CArG-containing 95-bp probe, addition of increasing amounts of rPTEN combined with rSRF resulted in a higher mobility shift with maximal concentrations of rPTEN forming a single higher mobility band compared with rSRF alone (Fig. 3c, lanes 3–6). No DNA–protein complex was observed when rPTEN alone was incubated with the 95-bp probe, indicating PTEN does not bind to this DNA under the conditions of the experiment (Fig. 3c, lane 7). Addition of saturating amounts of rSRF to the 95-bp DNA–rSRF reaction resulted in the formation of a single, lower mobility protein–DNA complex (Fig. 3d, lane 5) that had a gel shift similar to the slower migrating complex observed in the DNA–rSRF complex (band 2; compare with Fig. 3d, lane 2). In contrast, while addition of saturating amounts of rSRF to the DNA–rSRF–rPTEN reaction formed a single, lower mobility band compared with DNA–rSRF–rPTEN (band 1; Fig. 3d, compare lane 4 with lane 3), this complex exhibited a higher mobility compared with DNA plus saturating amounts of rSRF alone (Fig. 3d, compare band 1 with band 2). Western blotting of the EMSA was used to show that PTEN associates with SRF in this complex. An overlapping PTEN-specific band was observed in the DNA–rSRF–rPTEN lanes, but not DNA–rPTEN lanes (Supplementary Fig. 5b, compare lanes 3 and 4 right blot and upper graph); this complex has the same electrophoretic mobility as the DNA–rSRF–rPTEN EMSA complex (Supplementary Fig. 5b, compare lanes 3 both blots and lower graph). To confirm the presence of PTEN in the DNA–rSRF–rPTEN EMSA complex, supershift experiments were performed using a PTEN-specific antibody. Addition of a PTEN-specific antibody to the 95-bp DNA probe–rSRF–rPTEN reaction resulted in a complex shift of lower mobility compared with reaction without antibody (Fig. 3e, compare lane 4, band 3 with lane 3, band 1). As a positive control, a supershift was also observed with the addition of an SRF-specific antibody to the 95-bp DNA probe–rSRF–rPTEN reaction (Fig. 3e, compare lane 5, band 2 with lane 3, band 1). These results indicate that PTEN–SRF together form a higher order complex with DNA on SM promoter regions. Further, given the faster migration of the DNA–rSRF–rPTEN complex compared with DNA–rSRF alone, these data suggest that addition of PTEN to a larger multi-CArG-containing DNA–protein reaction likely promotes a conformational change of the protein–DNA complex.

**Figure 4:**
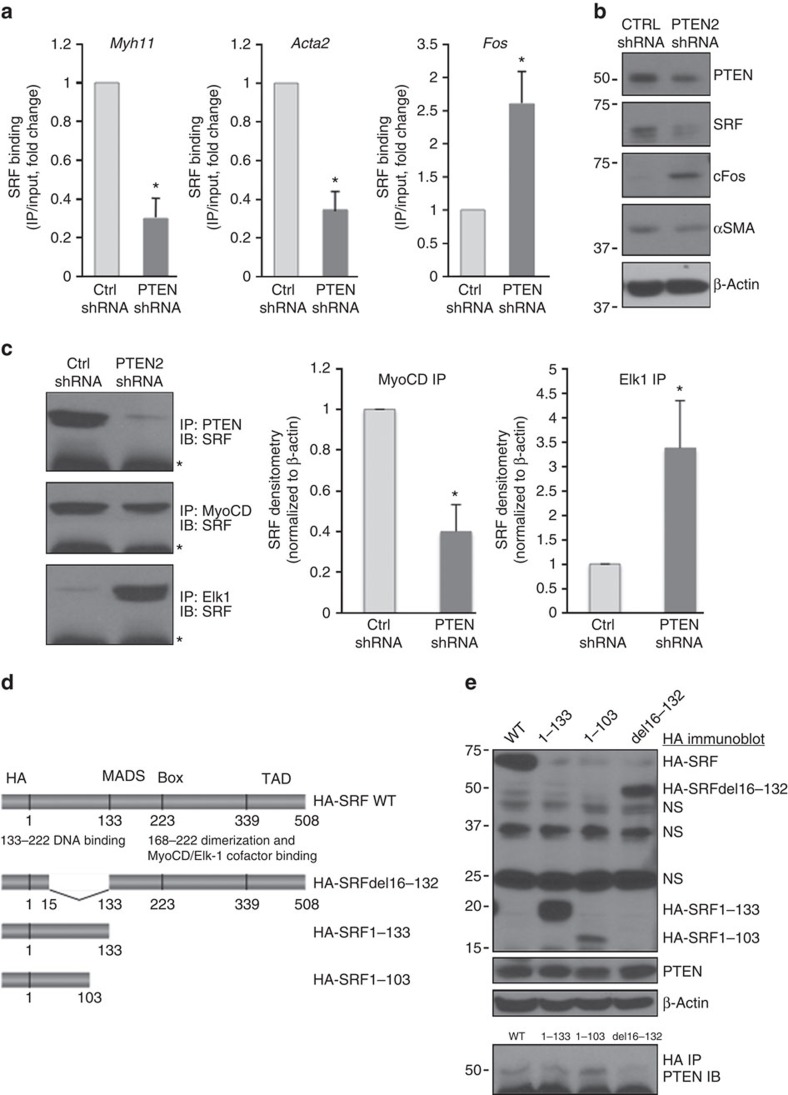
PTEN is essential for SRF binding to CArG boxes of SM genes. SMCs stably expressing control (Ctrl) or PTEN-specific shRNA were serum-restricted for 48 h. (**a**) ChIP assays for SRF binding to CArG elements in the *Myh11* (left), *Acta2* (middle) and *Fos* (right) promoters were performed as described in Fig. 3. Data represent average fold changes±s.e.m. *N*=3 independent experiments; *P<0.01 versus Ctrl shRNA. (**b**) SMCs stably expressing control (Ctrl) or PTEN-specific shRNA were serum-restricted for 48 h. WCL were analysed for total PTEN, SRF, c-fos, and αSMA. β-Actin was used as a loading control. (**c**) PTEN (top), Myocardin (MyoCD; middle) or Elk-1 (bottom) proteins were immunoprecipitated from WCL of SMCs serum restricted for 48 h. 10% of input WCL and co-immunoprecipitating SRF were detected by immunoblotting. Left—representative blots. Right—fold changes in densitometry measurements±s.e.m. for SRF–MyoCD and SRF–Elk-1.versus *N*=3; *P<0.01 versus Ctrl shRNA. *, heavy chain IgG. (**d**) Left—schematic diagram of wild-type HA-tagged SRF (top) and the mutant forms of HA–SRF used to map the PTEN binding domain. (**e**) Right—L929 fibroblasts were transiently transfected with expression plasmids encoding wild-type HA-tagged SRF or HA-tagged SRF deletion mutant proteins. Top blots—western blot showing expression of wild-type and mutant HA-tagged SRF proteins (anti-HA antibody) and endogenous PTEN (anti-PTEN antibody) used for co-immunoprecipitation assays. β-Actin was used as a loading control. Bottom blot—SRF proteins were immunoprecipitated from WCL with an anti-HA antibody and co-immunoprecipitating endogenous PTEN was detected with an anti-PTEN antibody. *, heavy chain IgG. Shown is a representative blot from three independent experiments. Molecular weight markers were cropped out for final SRF blots; please see Supplementary Fig. 8.

To determine if PTEN is essential for SRF binding to SM gene promoters, ChIP assays were conducted on control and PTEN-deficient SMCs. Compared with controls, SRF binding to CArG elements in the *Myh11* and *Acta2* promoters was markedly reduced in PTEN-depleted SMCs (Fig. 4a); this was associated with enhanced SRF binding to CArG elements of the *Fos* promoter (Fig. 4a). Greater SRF binding to the *Fos* promoter correlated to increased cFos protein expression in PTEN-depleted SMCs compared with controls (Fig. 4b). Myocardin selectively increases SRF binding to CArG elements in SM genes, while Elk-1 enhances SRF binding to the *Fos* promoter. Repression of SM gene transcription occurs at least in part through disruption of SRF–myocardin interactions thus promoting SRF–Elk-1 association^17^. Using co-IP assays, compared with control SMCs, SRF–myocardin interactions decreased, but SRF–Elk-1 interactions increased in PTEN-depleted SMCs (Fig. 4c), consistent with the ChIP and western blot data and loss of SRF-dependent SM gene transcription. Myocardin and Elk-1 compete with each other for a common binding site on SRF^17^. A series of SRF deletion mutants were used to map the domain responsible for PTEN–SRF interaction and determine if this site is distinct from the myocardin/Elk-1-binding domain (Fig. 4d). Deletion of the central MADS box, that consists of the SRF DNA-binding domain, SRF dimerization domain and myocardin/Elk-1-binding site, had no effect on PTEN–SRF interaction. In contrast, deletion of the N terminus amino acids 16 through 132 resulted in loss of PTEN–SRF interaction (Fig. 4e). Therefore, sequences within the N terminus of SRF and separate from myocardin/Elk-1 interaction sequences are necessary and sufficient for interaction with PTEN.

### PDGF blocks PTEN binding to CArG elements in SM genes

**Figure 5:**
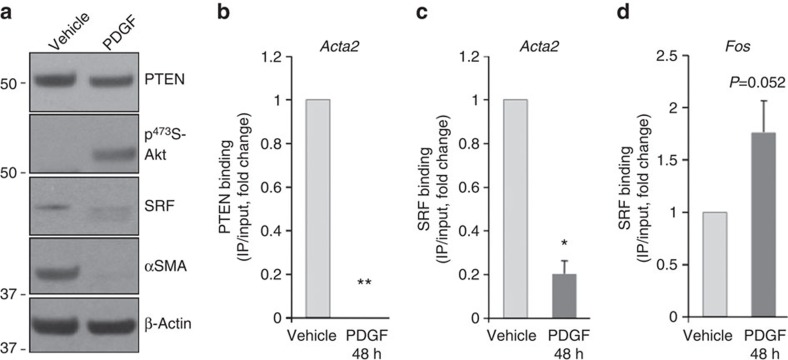
PDGF blocks PTEN and SRF binding to CArG elements in SM genes. SMCs were serum-restricted for 48 h followed by stimulation with vehicle control or 20 ng ml^−1^ platelet-derived growth factor-BB (PDGF-BB) for an additional 48 h. (**a**) Representative western blot for total PTEN, phospho-Akt, SRF and αSMA; *N*>3 independent experiments. (**b**–**d**) ChIP assays for PTEN (**b**) and SRF (**c**,**d**) binding to CArG elements in the *Acta2* (**b**,**c**) and *Fos* (**d**) promoters were performed as described above. Data represent average fold changes±s.e.m. *N*=3 independent experiments; unless otherwise noted, *P<0.01 versus vehicle control; ‘**' denotes statistical analysis not conducted due to complete loss of PTEN interactions in PDGF-stimulated SMCs (consistent undetectable values). Molecular weight markers were cropped out for final SRF blots; please see Supplementary Fig. 8.

PDGF-BB is a known physiological regulator of SMC phenotypic switching^5^ that represses SM gene transcription at least in part through regulating SRF binding to CArG boxes of SM genes^27^. We therefore investigated whether PDGF-BB regulated PTEN binding to SM promoters and therefore SM gene transcription. As shown in Fig. 5a, compared with vehicle control, PDGF-BB stimulation reduced expression of PTEN, SRF and αSMA. This was associated with loss of PTEN binding and reduced SRF binding to CArG boxes on SM gene promoters (Fig. 5b,c; *Acta2* shown), but increased SRF binding to *Fos* CArG boxes (Fig. 5d), similar to shRNA-mediated molecular depletion of PTEN and consistent with PDGF-mediated SMC dedifferentiation.

### PTEN-SRF interaction on CArG boxes of SM genes *in vivo*

**Figure 6:**
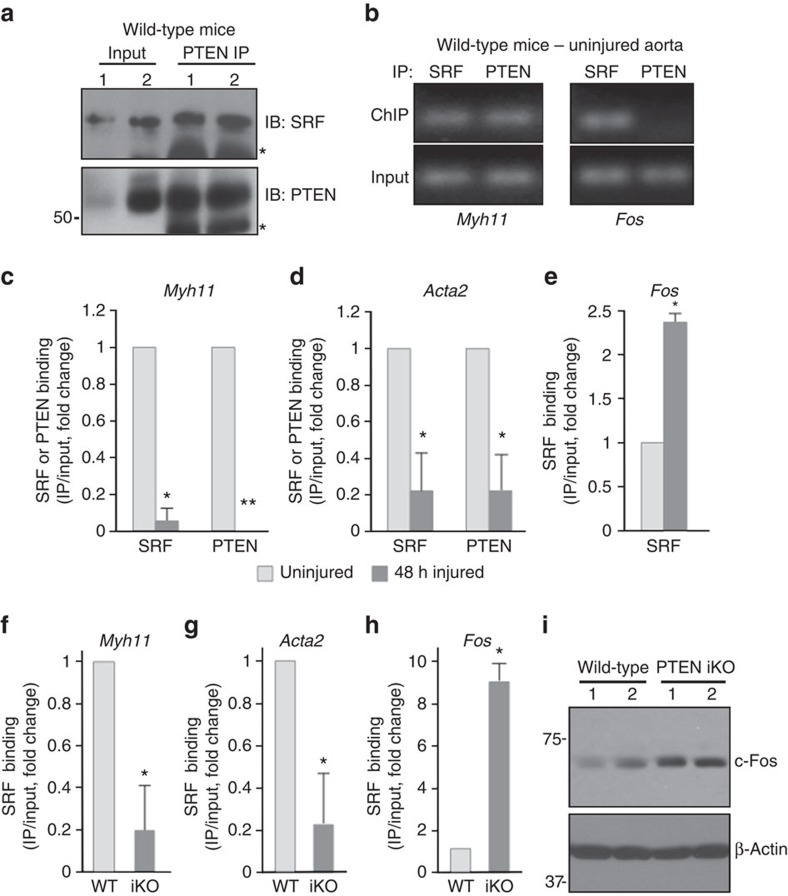
PTEN deficiency *in vivo* promotes loss of SRF binding to SM gene promoters. (**a**) PTEN was immunoprecipitated from WCL of smooth muscle-rich intact aortic media of wild-type mice. About 10% of input WCL and co-immunoprecipitating SRF were detected by immunoblotting. Representative blot showing co-IPs from two separate mice; *N*=6 independent mice analysed. *, heavy chain IgG. (**b**) ChIP assays for PTEN and SRF binding to CArG elements in the *Myh11* (left) and *Fos* (right) promoters were performed on intact aortic media using pooled arteries from five individual wild-type mice. (**c**–**e**) Mice underwent carotid artery ligation-induced injury as described in Methods. Chromatin was isolated from 48-h injured and contra-lateral uninjured arteries and analysed by ChIP for SRF (**c**–**e**) and PTEN (**c**,**d**) binding to CArG elements in the *Myh11* (**c**), *Acta2* (**d**) and *Fos* (**e**) promoters. Data represent average fold changes±s.e.m. *N*=3 independent experiments using pooled arteries from 9 to 11 individual mice; *P<0.01 versus uninjured control; ‘**' denotes statistical analysis not conducted due to complete loss of PTEN interactions in injured vessels (consistent undetectable values). (**f**–**h**) Smooth muscle-rich aortic media from wild-type (WT) and PTEN iKO (iKO) mice was analysed by ChIP for SRF binding to CArG elements in the *Myh11* (**f**), *Acta2* (**g**) and *Fos* (**h**) promoters. Data represent average fold changes±s.e.m. *N*=3 independent experiments using pooled arteries from five individual mice; *P<0.01 versus WT. (**i**) Western blot analysis for c-fos levels in whole cell lysates (WCL) of aortic media from WT or PTEN iKO mice. β-Actin was used as a loading control (western blot is from the same samples shown in Fig. 1c using the same β-Actin image). Representative blot from two mice per genotype with each lane representing an individual mouse. Molecular weight markers were cropped out for final SRF blot; please see Supplementary Fig. 8.

To establish the *in vivo* relevance of nuclear PTEN, we analysed SMC-rich aortic media from WT mice. Co-IP analyses revealed that PTEN and SRF interacted with each other (Fig. 6a), consistent with the *in vitro* cell data. ChIP analyses demonstrated that PTEN selectively interacted with CArG boxes of the *Myh11* promoter, but not the *Fos* promoter (Fig. 6b) in mature, uninjured aortic media. Vascular injury promotes SMC dedifferentiation through loss of SRF binding to SM genes^27^. To determine if injury-induced SMC dedifferentiation is associated with loss of PTEN binding to SM genes, we subjected mice to carotid artery ligation-induced injury. In agreement with previous findings^27^, compared with uninjured vessels, arterial injury assessed at 48 h resulted in reduced SRF binding to *Myh11* and *Acta2* CArG promoter regions (Fig. 6c,d), but enhanced SRF binding to *Fos* CArG elements (Fig. 6e). Importantly and consistent with a role for PTEN in facilitating SRF-dependent SM gene expression, injury resulted in complete loss (*Myh11*) or reduced (*Acta2*) PTEN binding to CArG boxes of SM gene promoters (Fig. 6c,d). To establish that nuclear PTEN is essential for SRF-dependent SM gene expression, ChIP assays were performed on aortic media from WT compared with PTEN iKO mice. Similar to vascular injury, SRF binding to CArG boxes in the *Myh11* and *Acta2* promoters was markedly reduced, but was increased on CArG elements of the *Fos* promoter in PTEN iKO mice compared with WT mice (Fig. 6f–h). Consistent with the *in vitro* data, enhanced SRF binding to the *Fos* promoter correlated to increased levels of c-Fos protein in aortic medial SMCs from PTEN iKO mice compared with WT mice (Fig. 5i). Collectively, these results reveal a novel function for nuclear PTEN as an essential regulator of SRF activity that is necessary for SM gene transcription. SMC PTEN loss promotes SRF co-factor switch, decreased SRF binding to SM gene promoters, but enhanced SRF interactions with *Fos* promoter elements.

### Loss of SM genes by PDGF is blocked by nuclear PTEN

**Figure 7:**
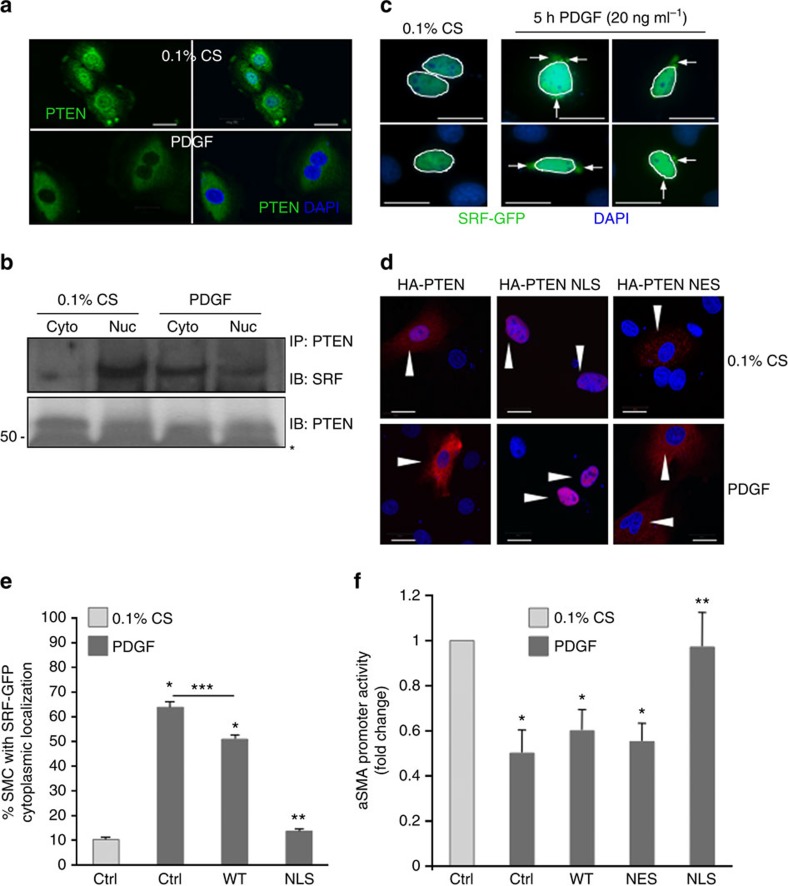
Nuclear PTEN blocks PDGF-mediated repression of SM gene transcription. (**a**,**b**) SMCs were serum-restricted for 48 h followed by stimulation with vehicle control or 20 ng ml^−1^ PDGF-BB for 24 h (**a**) or 48 h (**b**). (**a**) SMCs were fixed, immunofluorescently stained for PTEN (green) and analysed for PTEN localization using confocal microscopy; nuclei were stained for DAPI (blue). (**b**) PTEN was immunoprecipitated (IP) from cytoplasmic (cyto) and nuclear (nuc) fractions of vehicle- or PDGF-stimulated SMCs. Co-immunoprecipitating SRF was detected by immunoblotting (IB). Representative western blot from three separate experiments. (**c**) SMCs were transfected with a construct expressing SRF–GFP, maintained in serum-restricted conditions or stimulated with 20 ng ml^−1^ PDGF-BB, fixed and analysed for GFP localization; nuclei were stained for DAPI (blue). Shown are representative images (two serum-restricted and four PDGF-stimulated cells are shown); arrows indicate cytoplasmic localized SRF–GFP; nuclei are outlined with white lines. (**d**) SMCs were transfected with HA-tagged wild-type PTEN (WT), nuclear localized PTEN (NLS) or nuclear excluded PTEN (NES). SMCs were maintained in serum-restricted conditions or stimulated with 20 ng ml^−1^ PDGF-BB, fixed, immunofluorescently stained for HA (red) and analysed for PTEN localization; nuclei were stained for DAPI (blue). Arrowheads, HA–PTEN-transfected SMCs. (**e**) SMCs were transfected with GFP–SRF (ctrl) or co-transfected with GFP–SRF and WT PTEN or nuclear localized PTEN (NLS) then maintained in serum-restricted conditions or stimulated with 20 ng ml^−1^ PDGF-BB. Transfected SMCs exhibiting cytoplasmic GFP expression were scored as described in Methods. Data represent average per cent positive±s.e.m. *N*=3 independent experiments; *P<0.01 versus control 0.1% CS; **P<0.01 versus control PDGF and WT PTEN PDGF; ***P<0.01 versus control PDGF. (**f**) Non-transfected (Ctrl) and HA–PTEN-transfected SMCs were co-transfected with an *Acta2* promoter-Luciferase reporter construct. SMCs were serum-restricted for 48 h followed by stimulation with vehicle control or 20 ng ml^−1^ PDGF-BB for an additional 24 h. Luciferase activity normalized to β-galactosidase was determined; shown are fold changes from Ctrl serum-restricted (0.1% CS) SMCs. Data represent average fold changes±s.e.m. *N*=3 independent experiments; *P<0.01 versus Ctrl 0.1% CS; **P<0.01 versus Ctrl PDGF. Molecular weight markers were cropped out for final SRF blot; please see Supplementary Fig. 8. Scale bars for all images, 20 μm.

We next tested whether loss of nuclear PTEN plays a role in SMC phenotypic switching. PDGF treatment resulted in NES of PTEN (Fig. 7a), consistent with PDGF repression of SM gene expression and loss of SRF and PTEN binding to SM gene promoters (Fig. 5). Notably, cell fractionation and co-IP analyses showed that PDGF stimulation resulted in reduced nuclear, but increased cytoplasmic PTEN–SRF interactions (Fig. 7b). This was associated with translocation of a pool of SRF out of the nucleus in response to PDGF treatment (Fig. 7c). To test the significance of nuclear PTEN on SRF localization and SRF-dependent SM gene expression, WT SMCs were transfected with EV, haemagglutanin (HA)-tagged WT PTEN or HA-tagged WT PTEN harbouring artificial nuclear localization (NLS) or NES sequences. Under basal conditions, WT HA–PTEN localized to both the cytoplasm and nucleus, whereas NLS–HA–PTEN and NES HA–PTEN were enriched selectively in the nucleus or the cytoplasm, respectively (Fig. 7d). In response to PDGF, WT HA–PTEN was shuttled out of the nucleus, but NLS–HA–PTEN was retained in the nucleus (Fig. 7d). Co-transfection with green fluorescent protein-tagged SRF and WT PTEN demonstrated that a large percentage of SMCs overexpressing WT PTEN remain susceptible to PDGF-mediated SRF cytoplasmic shuttling (Fig. 7e). In contrast, overexpression of NLS–HA–PTEN blocked SRF nucleo-cytoplasmic translocation (Fig. 7e). Promoter-reporter assays were conducted by co-transfecting SMCs with the various PTEN constructs and an αSMA-luciferase reporter construct. As anticipated, PDGF treatment promoted loss of αSMA promoter activity in SMCs co-transfected with EV, WT HA–PTEN and NES HA–PTEN (Fig. 7f), consistent with PDGF-induced SMC dedifferentiation. Importantly, overexpression of nuclear localized PTEN prevented PDGF-dependent repression of αSMA promoter activity (Fig. 7f). Collectively, these data support the concept that nuclear PTEN prevents SRF nucleo-cytoplasmic translocation, and spatial regulation of a PTEN–SRF complex may be critical to control SMC dedifferentiation.

### Decreased PTEN expression in human atherosclerotic lesions

**Figure 8:**
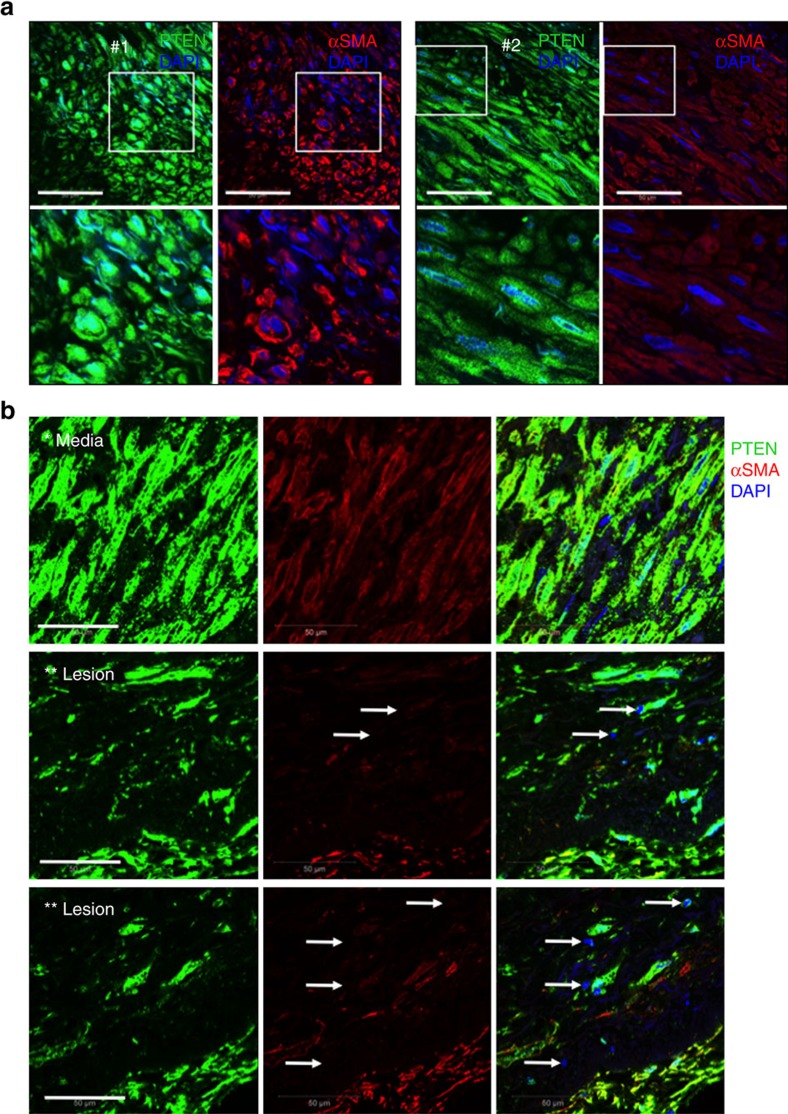
Loss of PTEN in intimal SMCs of human atherosclerotic coronary arteries. Double immunofluorescent staining for PTEN (green) and αSMA (red) was conducted on arterial tissues from human patients. (**a**) Confocal microscopic images of human coronary arterial media from two patients (#1 and 2); bottom panels are higher magnification images of boxed areas. Stains are shown separately rather than in overlay to demonstrate both cytoplasmic and nuclear PTEN staining, but exclusive cytoplasmic αSMA expression in normal medial SMCs. (**b**) Confocal microscopic images of right coronary artery with large atherosclerotic plaque (lower magnification H&E image is shown in Supplementary Fig. 7c; ‘*' and ‘**' represent the region of vessel shown in the H&E image). Top panels demonstrate cytoplasmic and nuclear PTEN staining in medial SMCs. Middle and bottom panels demonstrate loss of nuclear PTEN associated with loss of αSMA expression in plaque intimal SMCs (arrows). Right panels show merged images plus DAPI staining for cell nuclei. Scale bars for all images, 50 μm. H&E, haematoxylin and eosin.

A recent study used DNA microarray to identify differentially expressed genes in human atherosclerotic coronary arteries combined with meta-analysis to compare expression profiles of atherosclerotic coronaries with existing expression profiles from human atherosclerotic carotid arteries^56^. PTEN was identified among the common downregulated genes in human atherosclerotic coronary and carotid arteries. Therefore, to assess the relevance of nuclear PTEN to human atherosclerosis, we analysed atherosclerotic coronaries from a small cohort of patients compared with normal aorta and non-atherosclerotic control coronary arteries for expression of PTEN. By confocal immunofluorescent imaging, we found PTEN uniformly expressed in the cytoplasm and nucleus of aortic and coronary artery medial SMCs (Fig. 8 and Supplementary Fig. 6a–c). Compared with medial SMCs, confocal immunofluorescence demonstrated loss of nuclear PTEN that was associated with loss of αSMA expression in intimal SMCs of human atherosclerotic lesions (Fig. 8b and Supplementary Fig. 6d). In contrast, αSMA-rich intimal SMCs in fibrous cap regions of atherosclerotic lesions exhibited nuclear expression of PTEN (Supplementary Fig. 6e). Immunofluorescence and immunohistochemical analysis of a limited number of human tissues representing various stages of atherosclerosis suggested the extent of SMC PTEN loss correlates to lesion severity (Supplementary Figs 6f and 7), indicating atherosclerosis progression may involve a chronic deficiency of SMC PTEN and subsequent loss of SRF transcriptional activity.

## Discussion

Here we describe a novel and previously unknown function for nuclear PTEN as an indispensable regulator of SRF transcriptional activity and demonstrate that its interaction with SRF is independent of its phosphatase activity. PTEN's canonical function is to dephosphorylate PIP3, which antagonizes cytoplasmic PI3-kinase/Akt signalling events^39^,^40^,^44^,^49^, as well as function as a phosphatase for several defined protein substrates^46^,^47^,^57^; however, recent studies identified nuclear and phosphatase-independent functions for PTEN as well^48^,^50^,^58^. In the setting of cancer, NLS of PTEN is essential for its tumour suppressive function^52^,^53^ and a recent study demonstrated that PTEN interacts with chromatin^59^. However, to our knowledge our study is the first report demonstrating association of PTEN with a transcription factor on promoter elements critical for regulation of SM contractile genes. EMSA data using rPTEN and SRF suggest that PTEN does not bind to this DNA by itself, but rather associates with SRF to facilitate SRF-dependent transcription. Moreover, PTEN–SRF interactions favour selective DNA binding of SRF on SM gene promoters, but not SRF-dependent IEG promoters, suggesting a mechanism actively repressing SMC phenotypic switching. Inactivation of PTEN both in cultured SMCs and in *in vivo* mouse models results in loss of SRF binding to SM gene promoters, but enhanced SRF binding to proliferation-associated gene promoters demonstrating its critical role in maintenance of SMC contractile gene expression.

We demonstrate that physiological agents that drive SMC dedifferentiation (for example, PDGF) promote NES of PTEN and a pool of SRF, resulting in lower levels of nuclear SRF. This was associated with enhanced SRF activity on target genes involved in cell proliferation (for example, *Fos*). These findings are consistent with a previous study demonstrating enhanced SRF binding to the *Fos* promoter in response to PDGF and vascular injury^27^. While seemingly contradictory, the paradoxical regulation of differentiation- and growth-associated genes by SRF is complex and likely dependent on additional mechanisms not yet identified. Of potential interest, SRF, first identified as a regulator of IEG expression in fibroblasts, is found at much lower levels in fibroblasts compared with SMCs (Supplementary Fig. 4). Despite lower SRF expression, fibroblasts induce SRF-dependent c-Fos expression in response to growth signals. Combined with our ChIP data demonstrating loss of SRF binding to SMC promoters with PTEN loss, this could suggest that despite a reduction of SRF–PTEN NES results in an overall bioactive nuclear pool of SRF available to bind alternative promoters (for example, *Fos*). As decreased expression of PTEN was observed in intimal cells in atherosclerotic lesions from human patients, this process is implicated as a possible mechanism in vascular disease progression and underlies the important clinical relevance of our findings.

Since a nuclear localized PTEN construct blocked PDGF-mediated repression of αSMA expression, our data suggest that PDGF actively targets PTEN for NES as a mechanism to dynamically suppress SM gene expression. Alternatively, it remains possible that nuclear PTEN blocks PDGF-mediated repression of SM genes independent of a direct role on PTEN localization. PTEN does not contain classic nuclear import motifs^47^. Recent work identified two lysine residues that are mono-ubiquitinated to transport it between the nucleus and cytoplasm^52^ and studies in the setting of cancer demonstrated that mono-ubiquitination is critical to control NLS^53^. Once in the nucleus, PTEN is de-ubiquitinated by USP7 and subsequently remains nuclear localized. It will be of significant interest and the focus of our future studies to define whether alterations in USP7 activity and thus the mono-ubiquitination status of PTEN underlie SMC dedifferentiation and vascular disease progression, which could lead to novel therapeutics that selectively target this system. Alternative mechanisms of PTEN nucleo-cytoplasmic shuttling include simple diffusion, RAN-mediated nuclear import and the potential use of non-traditional NLS-like sequences^48^.

SMCs and cardiomyocytes express unique sets of SRF- and myocardin-dependent contractile proteins that confer their contractile ability. Despite common transcriptional machinery, why SM genes are not activated in cardiomyocytes remains an intriguing question. While our findings reveal a critical role for PTEN in maintaining SM contractile gene expression, association of SRF with PTEN in cardiomyocytes was only observed under hypertrophic conditions. This finding suggests that PTEN is dispensable for regulating physiological cardiac contractile genes, but possibly plays a role in pathological cardiac hypertrophy. Moreover, lack of interaction under physiological settings suggests that blocking PTEN–SRF nuclear association may be a unique mechanism repressing SM gene activation in cardiomyocytes. We propose this could be due to differences in nucleo-cytoplasmic trafficking of PTEN in cardiomyocytes or expression of a cardiomyocyte-specific co-repressor that prevents PTEN–SRF association with SM gene promoters. SRF-dependent induction of the cardiac ‘fetal' gene program is a characteristic response during pathological cardiac hypertrophy^60^. While beyond the scope of the current study, it will be of interest to determine whether PTEN regulates cardiac fetal genes in the setting of pathological cardiac hypertrophy and determine the role of agonist-induced PTEN nucleo-cytoplasmic shuttling in this context. Finally, while PTEN–SRF interaction was also detected in fibroblasts, PTEN overexpression to levels comparable in SMCs was not sufficient to activate SM genes. Ectopic expression of myocardin in fibroblasts, which normally lack myocardin, promotes SM gene induction; reviewed in refs 20, 22. As discussed by these authors, if additional co-factors other than SRF–myocardin are necessary for SM gene activation, they must be expressed by the fibroblasts. Our ongoing studies are analysing SM gene expression by myocardin overexpression in fibroblasts lacking PTEN, which will demonstrate the essential function of PTEN on SM gene transcription.

Regulated co-factor switching combined with the ability of SM gene DNA to appropriately bend have been proposed as potential mechanisms controlling which co-factor interacts with SRF to coordinate SM gene or IEG expression; reviewed in ref. 19. While our data suggest that PTEN does not specifically interact with DNA, results from our study demonstrate that PTEN and SRF together form a higher order protein–DNA complex. PTEN associates with the N terminus of SRF, a domain distinct from the myocardin- and Elk-1-binding domain. Therefore it is unlikely that there is direct competition between PTEN and Elk-1 for binding to SRF. In addition, using a longer, multi-CArG 95-bp DNA probe, intriguing EMSA results suggest that PTEN potentially promotes a conformational change in the PTEN–SRF–DNA complex, resulting in increased electrophoretic mobility under non-denaturing conditions compared with SRF–DNA alone. These data could imply a role for PTEN in altered DNA conformation that results in the stabilization of interactions or facilitation of contact between essential regulatory factors required for SM gene transcription (for example, SRF–myocardin). Our studies focused on αSMA and SM-MHC expression, two SM genes harbouring multiple CArGs in their promoter regions and previously shown to require cooperativity among the CArG boxes for activation of gene expression. Alternatively, we cannot rule out the possibility that PTEN alters SRF dimerization and/or DNA binding, which could result in the increased mobility. While we favour the former model, future studies will address these questions in more detail.

Co-IPs demonstrating PTEN–SRF interaction in the nucleus, ChIP data demonstrating that PTEN binds to CArG-containing chromatin, EMSA data using purified rPTEN and SRF demonstrating that PTEN directly interacts with SRF on CArG elements of SM genes, and loss of SRF binding to SM genes, loss of SRF–myocardin interaction and a functional loss of SMC contractility in the setting of PTEN deficiency, all hallmarks of SMC dedifferentiation, support the concept that PTEN serves as an SRF transcriptional co-factor. However, to demonstrate the importance of direct PTEN–SRF interaction for control of SMC gene transcription, attempts were made to show that WT SRF add-back, but not add-back using the non-binding SRF mutant (Fig. 4), restores αSMA promoter activity in SRF-depleted SMCs. Two independent investigators conducted a total of seven independent experiments using two separate SRF-depleted SMC cell lines with each experiment consisting of technical duplicates and experimental replicates to test various concentrations of DNA. We were unable to obtain reproducible data, implying that *in vivo* the interaction between PTEN and SRF may be indirect thereby ruling out a role for PTEN as a conventional transcriptional co-factor. It is possible that both technical challenges as well as potential biological reasons could explain our findings. For instance, while we demonstrated direct interaction between SRF and PTEN using recombinant proteins in an *in vitro* pull down assay (Fig. 2f), it is possible that direct interaction is not essential for PTEN's effect on SMC gene transcription and that other ‘bridging' proteins facilitate PTEN's effect. We also demonstrated that PTEN interacts with myocardin in whole cell lysates. Whether this is direct is yet to be determined, but is an area we are exploring.

Our studies suggest a phosphatase-independent effect of PTEN on SRF–PTEN interaction, which is supported by the following data: (1) Overexpression of phosphatase-dead PTEN in PTEN-reduced SMCs restores PTEN–SRF interactions; (2) SRF levels are not restored in PTEN-reduced SMCs by inhibition of Akt signalling; and (3) Overexpression of phosphatase-dead PTEN increases SRF and αSMA levels. Our previous data, however, supported a phosphatase-dependent effect of PTEN given that inhibition of Akt activity in SMCs expressing reduced levels of PTEN partially restored SM gene expression^37^. It is possible that phosphatase-dependent and -independent effects are not mutually exclusive. For instance, nucleo-cytoplasmic shuttling of PTEN could be dependent on Akt activity. In addition or alternatively, PTEN could function as a nuclear protein phosphatase that directly interacts with SRF and dephosphorylates sites required for SRF SM gene expression. Phosphorylation of SRF at S162 was shown to block binding to SM gene promoters while promoting binding to the *Fos* promoter, suggesting a novel switch-directing SM gene or IEG expression^61^. PTEN-mediated dephosphorylation of this site could serve as a means of maintaining SM gene transcription and would identify SRF as a novel protein target of PTEN. Thus, it is likely that PTEN possesses both phosphatase-independent (for example, interaction with SRF) and -dependent functions essential for regulating SM gene expression. Our ongoing studies are designed to clarify these findings in more detail.

**Figure 9:**
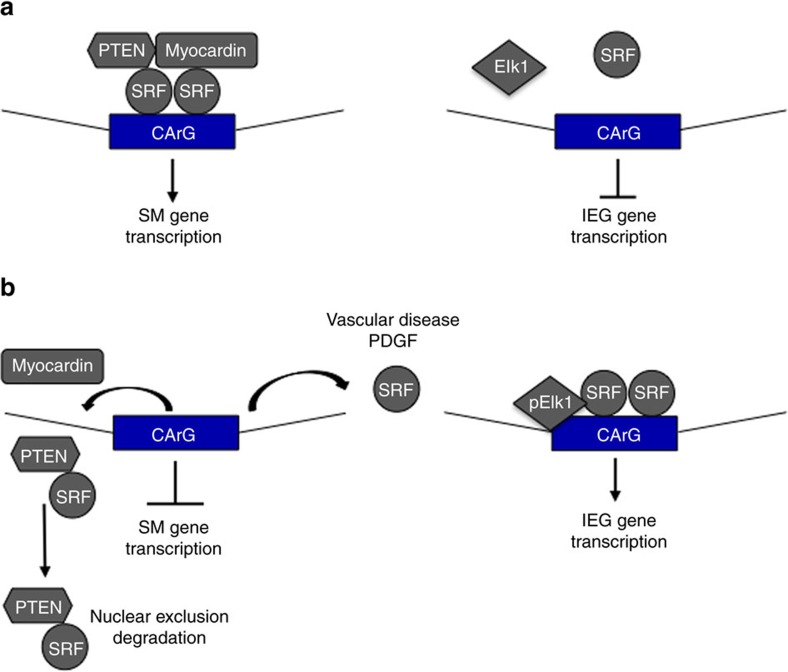
Proposed model of PTEN–SRF control of SMC differentiation. (**a**) PTEN, SRF and myocardin form a higher order transcriptional complex facilitating interaction of SRF on SM gene promoters, but not IEG promoters (for example, cFos) thus maintaining the SMC contractile gene expression. (**b**) Stimuli that promote SMC dedifferentiation (for example, vascular injury/disease, PDGF) result in loss of PTEN and SRF interaction on SM gene promoters, nuclear exclusion of PTEN and a pool of SRF, and transcriptional repression of the SM differentiation program. Loss of PTEN promotes SRF–cofactor switching and enhanced SRF binding to IEG promoters.

Our recent published work identified a miRNA network utilized by SRF to regulate PTEN-dependent SMC proliferation and inflammatory mediator production^37^. The current data support a positive feedback loop between PTEN and SRF that underlies maintenance of a contractile SMC phenotype (Fig. 9). Our data link loss of endogenous PTEN, which antagonizes multiple pathological pathways linked to vascular disease, as a major mechanism facilitating disease progression. In contrast to the many individual and likely redundant growth factors/cytokines found upregulated in diseased vessels, PTEN therefore represents a potentially significant therapeutic target. Activated SMCs contribute to lesion formation through proliferation, inflammatory cytokine production and abnormal vessel contractility. Thus, targeting PTEN–SRF nuclear interactions has the potential to develop novel therapeutics critical to preserve the mature differentiated SMC phenotype for such purposes as stabilization of atherosclerotic lesions, inhibition of in-stent restenosis and perhaps stabilization of a neovasculature in the setting of ischaemic tissue injury or tumour progression.

## Methods

### Cell culture and reagents

Primary rat aortic SMCs were isolated and cultured as previously described^35^. Briefly, the aggregate population of aortic medial SMC from adult Sprague Dawley rats was aseptically dissected and SMCs were obtained by digestion in Eagle's MEM Medium (EMEM) containing collagenase and elastase. Isolated cells were maintained in EMEM containing 10% calf serum (CS) and were used as primary cultured cells through passage 10. PTEN-depleted SMCs were generated using lentiviral shRNA plasmids (OpenBiosystems, AL), as described by us previously^35^. Infections using lentiviral particles expressing control or PTEN-specific shRNA were performed on primary rat aortic SMC (passage 3–6) according to protocols provided (OpenBiosystems, AL). Pools of infected cells were selected in 1.5 μg ml^−1^ puromycin (Sigma, CA) and used for experiments. SMC pools were screened by immunoblotting with PTEN antibodies as described below. Human primary aortic SMCs (ATCC PCS-100-012) were maintained in vascular cell basal medium (ATCC PCS-100-030; ATCC PCS-100-042) according to ATCC specifications. NRVMs were isolated and cultured as previously described^62^. Briefly, NRVMs were prepared from hearts of 1- to 3-day-old Sprague Dawley rats. Cells were cultured overnight on 10-cm plates coated with gelatin (0.2%; Sigma) in Dulbecco's Modified Eagle's Medium (DMEM) containing 10% CS, L-glutamine (2 mM) and penicillin–streptomycin. To promote hypertrophy, NRVMs were stimulated with 10 μM PE for 48 h. HEK 293 cells and L929 fibroblasts were obtained from ATCC (CRL-1573 and CCL-1, respectively) and cultured in DMEM containing 10% CS and penicillin–streptomycin. Trypsin/EDTA, Hank's Balanced Salt Solution (HBSS), MEM and DMEM were from Invitrogen, CA. Unless otherwise noted, chemicals and reagents were obtained from Sigma Chemical Co. (Sigma, MO). PDGF-BB (Upstate, Millipore) was used at 20ng ml^−1^, MG-132 (Calbiochem, Millipore, MA) was used at 20 μM, and LY294002 (Calbiochem, Millipore) was used at 20 μM.

### Animals

*Inducible smooth muscle-specific PTEN iKO mice*. PTEN^flox/flox^ mice (Dr Mak, Ontario Cancer Institute, the University of Toronto, Toronto, Ontario, Canada) and smooth muscle myosin heavy chain (*Myh11*)-CreER^T2^ transgenic mice (Dr Offermanns, the University of Heidelberg, Heidelberg, Germany) were bred to generate tamoxifen-inducible SMC-specific PTEN iKO mice, as described by us previously^38^. Controls expressed *Myh11*-CreER^T2^ but were WT for PTEN. Only male mice were used for these studies as the transgene for the *Myh11*-CreER^T2^ mice inserted on the ‘Y' chromosome. PTEN iKO and WT mice were fully backcrossed to a C57BL/6 background. Three-month-old mice received 1 μg i.p. tamoxifen injections (suspended in corn oil) for 5 consecutive days 1 week before they were killed and aortas were harvested for contractility experiments, western immunoblot analysis or ChIP analysis.

*Carotid artery ligation injury*. Four-month-old male FVB mice were used for vascular injury experiments. Left carotid arteries were isolated and completely ligated just proximal to the carotid bifurcation. Two days following injury, right unligated and left ligated carotid arteries were harvested, cross-linked in formaldehyde and processed for ChIP, as described below. Mice for all experiments were maintained in the Center for Comparative Medicine, and procedures were performed under compliance with ethical regulations and approved by the Institutional Animal Care and Use Committee at the University of Colorado Denver.

### Contractility assay

Maximal force generation in response to KCl was examined to determine electromechanical coupling changes, responses to PE was examined to determine pharmacomechanical coupling defects and vessels were examined to determine maximal Ca2+-induced vasoconstriction as a measure of Ca2^+^ sensitivity of myofilaments, as described previously^63^. Briefly, aortic rings were excised, placed in ice-cold physiological saline, and debrided of loose fat and connective tissue as previously described. Tissues were mounted between two minutien pins; one fixed and the other attached to a force transducer (AE 801, AME, Horten, Norway) and incubated in a well on a bubble plate for measurement of isometric force. Aortic rings (⩾12 tissues for each condition) were contracted with 108 mM KCl for two contraction/relaxation cycles or until reproducible forces were obtained. Isometric force was normalized to tissue cross-sectional area. Arterial responses were measured in response to both depolarization (KCl response) and agonist (4.75 mM KCl+20–2,000 nM PE); PE was tested in the presence of KCl to avoid transient contractions induced by PE alone and to ensure consistent levels of contraction. Ca2+ (0.1–1 μM) concentration–force relationships were obtained in a cumulative manner. Data were obtained with PowerLab hardware and analysed using Chart software.

### Western blotting

SMCs were lysed with ice-cold mPER lysis buffer (Thermo Fisher, MA) and protease inhibitors. Solubilized proteins were centrifuged at 14,000*g* (4 °C) for 10 min. Supernatants were separated using 10% SDS–polyacrylamide gel electrophoresis and transferred to Immobilon P membranes (Millipore, MA). Membranes were blocked for 1 h at room temperature in Tris-buffered saline (10 mM Tris-HCl, pH 7.4, 140 mM NaCl) containing 0.1% Tween-20 (TTBS) and 5% BSA (Sigma), and then incubated with 5% BSA in TTBS solution containing primary antibodies for 12–16 h at 4 °C. Membranes were washed in TTBS, and bound antibodies were visualized with alkaline phosphatase-coupled secondary antibodies and Lumi-Phos WB (Pierce, IL) according to the manufacturer's directions. Antibodies used: PTEN (1:1,000; Cell Signaling, MA), phosphoSer^473^Akt (1:1,000; Cell Signaling), total Akt (1:1,000; Cell Signaling), SM-MHC (1:100; Biomedical Technologies, MA), HA (1:100; Santa Cruz Biotechnologies, CA), cFos (1:100; Santa Cruz Biotechnologies), SRF (1:50; Santa Cruz Biotechnologies), α-SMA (1:10,000; Sigma), Elk-1 (1:100; Santa Cruz Biotechnologies), β-actin (1:20,000; Sigma), β-tubulin (1:500; Santa Cruz Biotechnologies), Lamin A/C (1:500; Cell Signaling) anti-Rabbit light chain specific (Cell Signaling), horseradish peroxidase (HRP)-anti-mouse secondary (Cell Signaling), and AP-anti-Rabbit secondary, and HRP-anti-Rabbit secondary (Santa Cruz Biotechnologies). Changes were quantitated by densitometry using Image J software (rsb.info.nih.gov). Full uncropped western blots can be found in Supplementary Fig. 8.

### Quantitative RT–PCR

Total RNA was isolated from SMCS using the QIAshredder and RNeasy Plus kits (Qiagen). First strand complementary DNA (cDNA) was made using the iScript cDNA synthesis kit (Bio-Rad). Sequence-specific primers were designed using the MacVector program (Macvector, NC): SRF: sense (5′- tctcaggcaccatccaccat -3′), antisense (5′- cccagcttgctgtcctatcac -3′); β-actin: sense (5′- agggtgtgatggtgggtatgg -3′), antisense (5′- aatgccgtgttcaatgggg -3′). Quantitative real-time PCR was performed as previously described^35^,^37^ and β-actin was used for normalization.

### Cytoplasmic/nuclear fractionation

SMCs were fractionated using the cytoplasmic/nuclear fractionation kit from Activ Motif with minor modifications to the manufacturer's protocol. Cells were washed twice with phosphatase buffer. Cytoplasmic fraction of SMCs was isolated with hypotonic buffer, but without adding detergent. The nuclear and membrane fraction was pelleted by centrifugation, and lysis buffer was added to isolate the nuclear fraction. The membrane fraction was pelleted and removed. Fractionation efficiency was determined by probing for β-tubulin for the cytoplasmic fraction, and Lamin A/C for the nuclear fraction.

### Bacterial expression of rPTEN and SRF

*GST–SRF*. *E. coli* Rosetta (DE3) pLysS cells were transformed with the pGEX-5 × -3 plasmid carrying the GST–SRF gene. An overnight culture was grown in terrific broth (supplemented with 0.1% glucose in the presence of 34 μg ml^−1^ chloramphenicol and 100 μg ml^−1^ ampicillin). About 1% of the pre-culture was used to inoculate 500 ml of terrific broth media supplemented with 0.1% glucose and 100 μg ml^−1^ ampicillin. Bacterial cells were grown to an OD_600_ of 1.3 at 37 °C. The cells were chilled on ice to lower the temperature to 18 °C prior to induction by addition of IPTG (final concentration 0.5 mM) and then incubated for 2.5 h. The cells were pelleted at 10,000*g* for 20 min, flash-frozen, and stored at −80 °C. The GST–SRF purification was carried out at 4 °C. Cells were lysed using sonication in lysis buffer containing 20 mM HEPES, pH 7.5, 150 mM NaCl, 1 mM DTT, 10% glycerol, 0.01% TTBS with a protease inhibitor cocktail tablet (Roche, EDTA-free) and 0.2 mM PMSF. The insoluble fraction was cleared via centrifugation at 40,000*g* for 60 min. The soluble fraction was mixed with GST beads that were pre-equilibrated with lysis buffer. The beads were washed with 500 μl of lysis buffer and resuspended in 15 ml of lysis buffer. About 5 μl of a 1 mg ml^−1^ solution of Factor Xa (New England Biolabs) was added to the bead solution and left at 4 °C to cleave SRF from the beads. The protein was eluted from the beads, concentrated and stored at −80 °C until further use.

*His_6_-PTEN*. *E. coli* cells transformed with pQE30-His_6_-PTEN were grown at 37 °C to an OD_600_ of 1.0 in Luria Bertani broth (LB) containing 100 μg ml^−1^ ampicillin. Protein expression was induced by addition of IPTG to a final concentration of 0.5 mM at 18 °C and incubation for 4 h. The cells were harvested at 10,000*g* for 20 min, flash-frozen and stored at −80 °C. The His_6_-PTEN purification was carried out at 4 °C. Cells were lysed using sonication in lysis buffer containing 50 mM sodium phosphate, pH 8.0, 5 mM BME, 500 mM NaCl, 20 mM imidazole, 0.01% TTBS and a protease inhibitor cocktail tablet. The insoluble fraction was cleared via centrifugation at 40,000*g* for 60 min. The soluble fraction was mixed with Ni-NTA agarose beads that were pre-equilibrated with lysis buffer for 2–4 h. The beads were washed with 500 ml of lysis buffer and the protein was eluted with elution buffer (50 mM sodium phosphate, pH 8.0, 5 mM BME, 300 mM NaCl, 250 mM imidazole, 0.01% Tween-20). The protein was dialyzed into 20 mM sodium phosphate, pH 7.8, 1 mM DTT, 150 mM NaCl, 5% glycerol, 0.01% Tween-20 and stored at −20 °C until further use.

### Co-IP assay

SMCs were lysed with ice-cold mPER lysis buffer and protease inhibitors. Similarly, media from aorta was digested in mPER lysis buffer using m-tubes (Miltenyi Biotec, Germany). Solubilized proteins were centrifuged at 14,000*g* in a microcentrifuge (4 °C) for 10 min. Samples were immunoprecipitated with equal concentration and volumes. Samples were pre-cleared with 50 μl of a 50% slurry of protein A–Sepharose resin (Sigma) and rabbit IgG for 1 h at 4 °C on an end-over-end tumbler. After incubation the resin from each sample was pelleted, and supernatant was transferred to new tubes and incubated with αPTEN 1:50 (Santa Cruz Biotechnologies), αSRF 1:50 (Santa Cruz Biotechnologies), αMyocardin 1:40 (Santa Cruz Biotechnologies), αElk-1 1:40 (Santa Cruz Biotechnologies), αVCAM 1:50 (Santa Cruz Biotechnologies) or rabbit IgG for 16–18 h, 4 °C, on an end-over-end tumbler. After incubation, 45 μl of a 50% slurry of protein A-Sepharose resin was added and allowed to incubate for 1 h at 4 °C on end-over-end tumbler. Resin from each sample were then washed 3 × with mPER Buffer and the bound proteins were eluted with 2% SDS–DTT sample buffer and detected by western immunoblotting. For *in vitro* co-IP assays, rPTEN and SRF were incubated with 50 μl of a 50% slurry of protein A-Sepharose resin for 1 h at 4 °C on an end-over-end tumbler in Tris Buffer (10 mm Tris-HCl, pH 8.0, 100 mm NaCl, 10% glycerol, 2 mm MgCl_2_, 1 mm EDTA, 1 mm DTT and 100 μg ml^−1^ bovine serum albumin). After incubation, the resin from each sample was pelleted, and supernatant was transferred to new tubes and incubated with αPTEN 1 μg (Santa Cruz Biotechnologies), αSRF 1 μg or Rabbit IgG 1 μg (Millipore) 16–18 h, 4 °C, on an end-over-end tumbler. After incubation, 45 μl of a 50% slurry of protein A-Sepharose resin was added to each sample and allowed to incubate for 1 h at 4 °C on an end-over-end tumbler. Resin from each sample was then washed 4 × with Tris Buffer and a tube transfer before the last wash. The bound proteins were eluted with 2% SDS–DTT sample buffer and detected by western blotting.

For SRF truncation mutation co-IP experiments, L929 fibroblasts were transiently transfected with Lipofectamine PLUS (Invitrogen) according to the manufacturer's protocol. After 24 h of recovery, L929 were differentiated for 24 h in 2% FBS DMEM media to promote differentiation. Plasmid-encoding human SRF tagged with HA was obtained from Addgene (#119977). SRF truncation mutations were made using the Quickchange II XL mutagenesis kit (Agilent Technologies) according to the manufacturer's instructions. The mutations were made using the following primers SRF 1-133 (5′- cttcttacccggcttctacccgctcaccgcgcc -3′; 5′- ggcgcggtgagcgggtagaagccgggtaagaag -3′), SRF 1-103 (5′- ccgatctccatctagctcaggctccgc -3′; 5′- gcggagcctgagctagatggagatcgg -3′), SRF del16-132 (5′- gcttggccccgccccggccc -3′; 5′- gggccggggcggggccaagc -3′). co-IP was performed as above with αHA 1:33 (Santa Cruz Biotechnologies).

### Electrophoretic mobility shift assay

Oligonucleotides used in EMSAs were designed based on work published by Mack *et al*.^55^ and purchased commercially (Integrated DNA Technologies, IA): CArG B, 5′- GAGGTCCCTATATGGTTGTG -3′ and 95 bp 5′- AATTCTGGGGTGTATCTTGCCCTATATGGGATATCGCTCTGACCGTTAACTAGGACTAGCT GAGGGTAGCCTTGTTTGGCTGCATCTGTTTCTTTTCGGCTAGC -3′. DNA was synthesized with fluorescein (IDT) and purified using DEAE anion exchange chromatography and a gradient of 0–1 M NaCl in standard TE buffer. Protein–DNA-binding reactions were performed in 1 × binding buffer (10 mM Tris pH 8.0, 1 mM EDTA, 8% glycerol) in a 10 μl reaction volume that included the following; ∼150 ng of fluorophore-labelled DNA, either 1–3 μl of rSRF purified protein and/or 1–10 μl of rPTEN purified protein. After 1 h incubation at room temperature, the samples were subjected to electrophoresis on a non-denaturing 4–20% gradient Tris-glycine polyacrylamide gel (pre-cast; Bio-Rad), pre-run at 75 V for 15 min. Electrophoresis was performed at 75 V in 1 × Tris-glycine running buffer pH 8.3 at room temperature. Gels were imaged using a Li-Cor Odyssey Fc imager. After imaging, gels were transferred to PVDF membrane for western blot analysis with PTEN antibody. Changes were quantitated by densitometry using Image J software (rsb.info.nih.gov).

### Quantitative ChIP

ChIP was performed on cultured cells, mouse media layer from aortas and mouse carotid arteries. Animals were killed, perfused with 1 × PBS plus heparin, vessels dissected and washed in ice-cold HBSS to remove blood and debris. Isolated aortic media or whole carotid arteries were then snap frozen in liquid nitrogen and stored at −80 °C. The tissues were later crushed in liquid nitrogen with mortar and pestle, transferred directly to 37 °C 1% formaldehyde in MEM solution for 10 min at 37 °C, washed three times with ice-cold PBS and taken through the rest of the ChIP protocol provided with the kit (Millipore). Briefly, samples were lysed with SDS, sonicated with a Branson Digital Sonifier 450 (Branson, CT) for 35–45 s with 5-s increment pulses and centrifuged at 14,000*g* for 10 min at 4 °C. Samples were then analysed on 1% agarose gels for concentration, and volumes were normalized with SDS buffer. ChIP was performed with αPTEN, αSRF or αElk-1. DNA was purified with QIAquick PCR purification kits (Qiagen,). Real-time PCR with Power SYBR Green mastermix (Life Technologies, CA) was used to analyse ChIP. For each primer set, the ratio of IP to Ref was calculated using the formula: 2^Ct(Ref)–Ct(IP)^–2^Ct(Ref)–Ct(no-antibody control)^ based on previously described methods^64^. Data from a minimum of three independent experiments were averaged and s.e. of the mean were calculated. Primer sequences: *Acta2* forward, 5′- AGCAGAACAGAGGAATGCAGTGGAAGAGAC -3′; reverse, 5′- CCTCCCACTCGCCTCCCAAACAAGGAGC -3′; *Fos* forward, 5′- CGGTTCCCC CCCTGCGCTGCACCCTCAGAG -3′; reverse, 5′- AGAACAACAGGGACCGGCCGTGGAAACCTG -3′; *Myh11* forward, 5′- CTGCGCGGGACCATATTTAGTCAGGGGGAG -3′; reverse, 3′- CTGGGCGGGAGACAACCCAAAAAGGCCAGG -5′; . PCR conditions were as follows: 15-s denaturation at 95 °C, 60-s annealing at 65 °C and 45-s extension at 72 °C (40 cycles).

### Plasmids, transient promoter assays and adenovirus transduction

For recombinant protein expression, SRF–GST plasmid was generated in pGEX-5X-3 (Amersham) and PTEN-His vector was a gift from Dr Yamada^57^. WT, NLS and NES HA–PTEN, PTEN G129R and PTEN G129E plasmids were obtained from Addgene (#10750, #10933, #10932, # 10748, #10746, respectively). αSMA promoter-luciferase reporter and CMV-β-galactosidase have been described previously^30^. For transient transfections for measuring αSMA promoter activity in response to altered PTEN localization, SMCs were transiently transfected with 5 μg of a luciferase vector regulated by 765 bp of the rat αSMA promoter, together with 1 μg of a plasmid-encoding CMV-β-galactosidase (Clontech, CA) for normalization of transfection efficiency, as well as, either 4 μg WT PTEN vector, NES PTEN vector, NLS PTEN vector or a control vector. SMCs, HEK 293 cells and L929 fibroblasts were transiently transfected with Lipofectamine PLUS according to the manufacturer's protocol. Plasmid-encoding human SRF tagged with GFP (RG208596) was obtained from OriGene. SMCs co-transfected with SRF–GFP and PTEN localization mutants were performed with Viafect transfection reagent (E4982) (Promega, WI). For PTEN overexpression experiments, SMCs were transduced in suspension with an EV adenovirus or adenoviruses expressing WT PTEN or phosphatase-inactive mutant PTEN (100 multiplicity of infection). Cells were then plated in EMEM with 10% CS. After 24 h, the SMCs were placed in fresh media containing 0.1% FCS for 24 h, whole cell lysates were harvested and analysed by western blot for expression of the indicated proteins.

### Immunofluorescence and confocal microscopy

SMCs were transiently transfected with 4 μg of WT PTEN vector, NES PTEN vector, NLS PTEN vector or a control vector, as described above. Transfected SMCs were plated on glass chamber slides, growth-arrested in Eagle's MEM containing 0.1% FCS for 24 h, followed by stimulation with PDGF-BB (20 ng ml^−1^) for 24 h. Cells were fixed in 4% PFA, permeabilized with MeOH and incubated with α-HA 1:50 (Santa Cruz Biotechnologies) or PTEN 1:50 (Novus, CO) antibody. Antigen/antibody complexes were visualized using Alexa Fluor-488 or -568-coupled secondary antibodies (Molecular Probes, NY). Coverslips were mounted with VectaShield medium containing DAPI to detect all cell nuclei (Vector Laboratories, CA) and cells imaged using a laser-scanning confocal microscope (510 META NLO, Carl Zeiss, Thornwood, NY) with a × 63 oil immersion objective. Images were analysed using LSM 510 software. For SRF–GFP localization experiment, SMCs were transiently transfected with 0.5 μg WT PTEN vector, NES PTEN vector or a control vector, and 1 μg of SRF–GFP. Transfected SMCs were plated on glass chamber slides or 60 mm dishes, transfected, growth-arrested in Eagle's MEM containing 0.1% FCS for 24 h, followed by stimulation with PDGF-BB (20 ng ml^−1^) for 5 h. Experiments conducted on 60 mm dishes were analysed for SRF–GFP localization at 5 h with live cell imaging. Experiments conducted on glass chamber slides were fixed in 4% PFA and mounted with VectaShield medium containing DAPI to detect all cell nuclei. Cells were visualized using a Nikon inverted fluorescence microscope equipped with NIS element-BR software.

### Human tissue sections and immunohistochemistry/fluorescence

Arterial tissues were obtained from explanted hearts from nine patients recruited as subjects for heart transplantation at the University of Colorado Anschutz Medical Campus. Tissues were collected under the Human Heart Tissue Bank protocol (COMIRB 01-568 PI P. Buttrick, Chief Cardiology Division; co-I for vascular studies K. Moulton). All appropriate informed consent was received from subjects and confirmation was obtained that they were aware that their samples would be used in research. For artery harvest, immediately after explant, left and right, left anterior descending, left marginal, and left circumflex coronary arteries as well as aortic tissues were dissected from diseased hearts, cleaned of fatty and cardiac muscle tissues, and processed for histology; a total of 24 individual vessels were analysed. After histological processing and haematoxylin and eosin staining, vascular sections were reviewed by Dr Moulton and characterized based on relative size of atheroma, presence of plaque neovascularization, composition of cells, lipid and matrix and media attenuation. De-coded and de-identified arterial tissues sections were obtained from Dr Moulton and were immunohistochemically or immunofluorescently stained for PTEN; patient clinical data were not available for these tissues. For immunohistochemistry, formalin-fixed, paraffin-embedded tissues were deparaffinized, rehydrated and underwent antigen retrieval by heating for 20 min at 100 °C in a decloaking chamber (Biocare). Antigen/antibody complexes were visualized using kits from Vector Laboratories and sections lightly counterstained with haematoxylin. Sections were imaged using an Olympus BX41 microscope equipped with SPOT software. Antibody used was monoclonal anti-PTEN (1:100; Cascade Bioscience, Winchester, MA). For immunofluorescent double staining, tissues were pre-treated as described above and sequentially incubated with a polyclonal anti-PTEN antibody (1:50; Millipore) followed by incubation with a cy3-conjugated monoclonal anti-αSMA antibody (Sigma). To detect PTEN expression, a TSA Biotin amplification system was used (Perkin Elmer). Briefly, following incubation with anti-PTEN, sections were sequentially incubated with secondary biotinylated anti-rabbit IgG (1:400), HRP-conjugated Streptavidin (1:100), BiotinylTyramide (1:50) and Alexa-488-conjugated Streptavidin. Sections were imaged using a laser-scanning confocal microscope (510 META NLO) and analysed using LSM 510 software.

### Statistical analysis

Data were expressed as means±s.e. and were determined using two-tailed *t*-test analysis. *P* values <0.05 were considered statistically significant. One-way analysis of variance (ANOVA) was performed to compare PTEN (and variants) transiently transfected SMC with the additional condition of PDGF-BB treatment. *P* values <0.05 were considered significant for the initial ANOVA and Tukey's multiple comparison test was then used (*P*<0.05).

## Additional information

**How to cite this article:** Horita, H. *et al*. Nuclear PTEN functions as an essential regulator of SRF-dependent transcription to control smooth muscle differentiation. *Nat. Commun.* 7:10830 doi: 10.1038/ncomms10830 (2016).

## Supplementary Material

Supplementary Information Supplementary Figures 1-8.
