# Supplementary material for: Nuclear PTEN functions as an essential regulator of SRF-dependent transcription to control smooth muscle differentiation
Source: Nat Commun. 2016 Mar 4;7:10830. doi: 10.1038/ncomms10830 (PMC5411712; doi:10.1038/ncomms10830)
Supplement: Supplementary Information — Supplementary Figures 1-8. [file ncomms10830-s1.pdf]

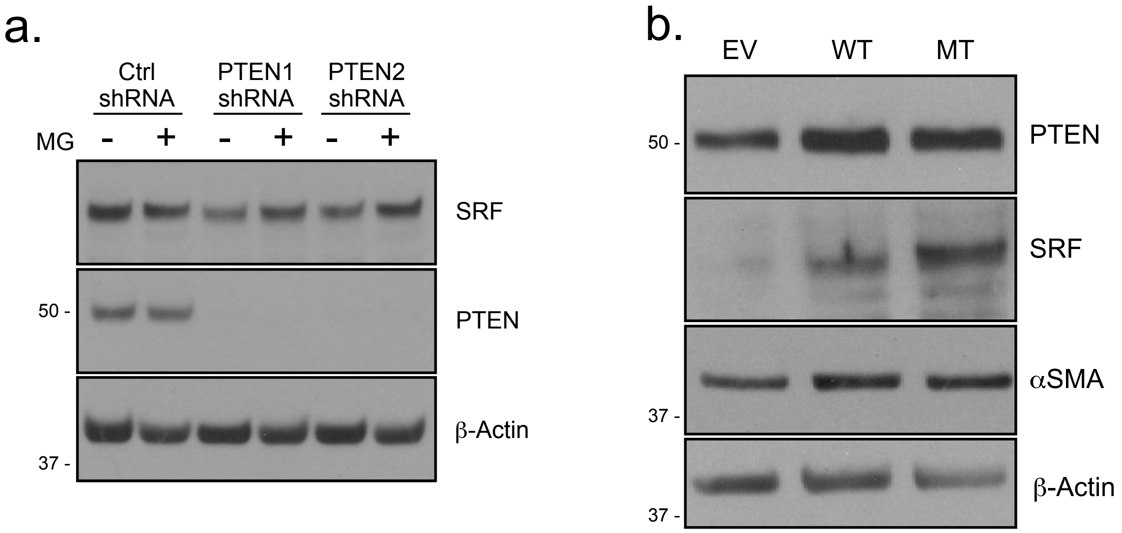

Supplementary Figure 1. SRF expression is independent of PTEN's phosphatase activity, but is dependent on proteasomal degradation **(a).** Ctrl and PTEN-deficient SMCs were serum-restricted in the presence or absence of the proteasome inhibitor, MG-132 (20 μM). WCL were analyzed for PTEN and SRF levels. β-actin was used as a loading control; shown are representative westerns from N≥3 independent experiments. **(b).** SMCs were transiently transduced with empty vector adenovirus (EV) or adenoviruses encoding wild-type PTEN (WT) or phosphatase inactive PTEN (MT) (MOI = 100). Cells were maintained in 0.1% FCS and WCL analyzed for PTEN, SRF, and αSMA levels. β-actin was used as a loading control; shown is a representative western of N≥3 independent experiments. Molecular weight markers were cropped out for final SRF blots; please see Supplementary Figure 8.

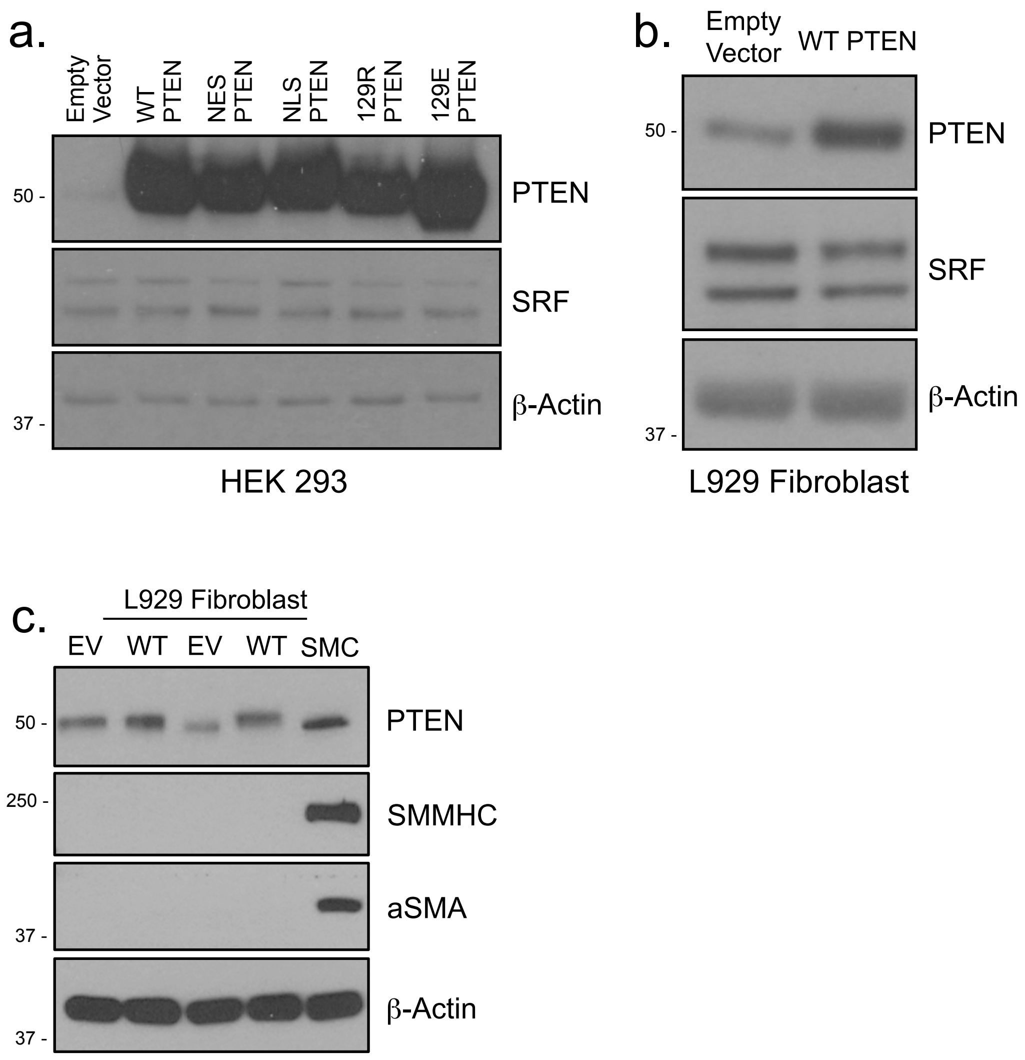

Supplementary Figure 2. PTEN does not regulate SRF in HEK 293 cells and L929 fibroblasts
(a). HEK 293 cells were transfected with empty vector or various PTEN constructs. WT: wild type; NES: nuclear excluded; NLS: nuclear localized; 129R: pan phosphatase dead; 129E: lipid phosphatase dead. WCL were analyzed for PTEN and SRF levels; β-actin was used as a loading control. **(b&c).** L929 fibroblasts were transfected with empty vector or WT PTEN. WCL were analyzed for PTEN, SRF, SMMHC, or αSMA levels; β-actin was used as a loading control. Panel (c) shows duplicate samples per condition for L929 cells. Molecular weight markers were cropped out for final SRF blots; please see Supplementary Figure 8.

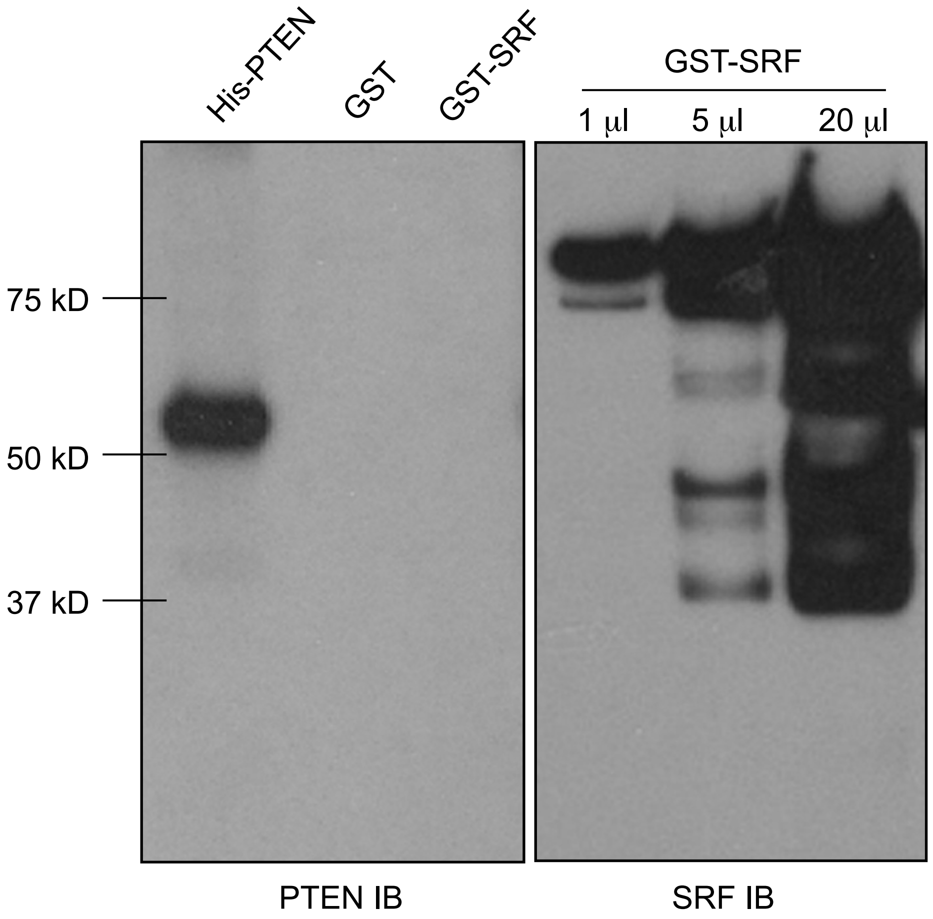

Supplementary Figure 3. Purification of recombinant His-PTEN and GST-SRF
Recombinant His-PTEN (left) and GST-SRF (right) were expressed and purified as described in Material and Methods. Purified proteins were analyzed by western blotting for PTEN (left panel) and SRF (right panel).

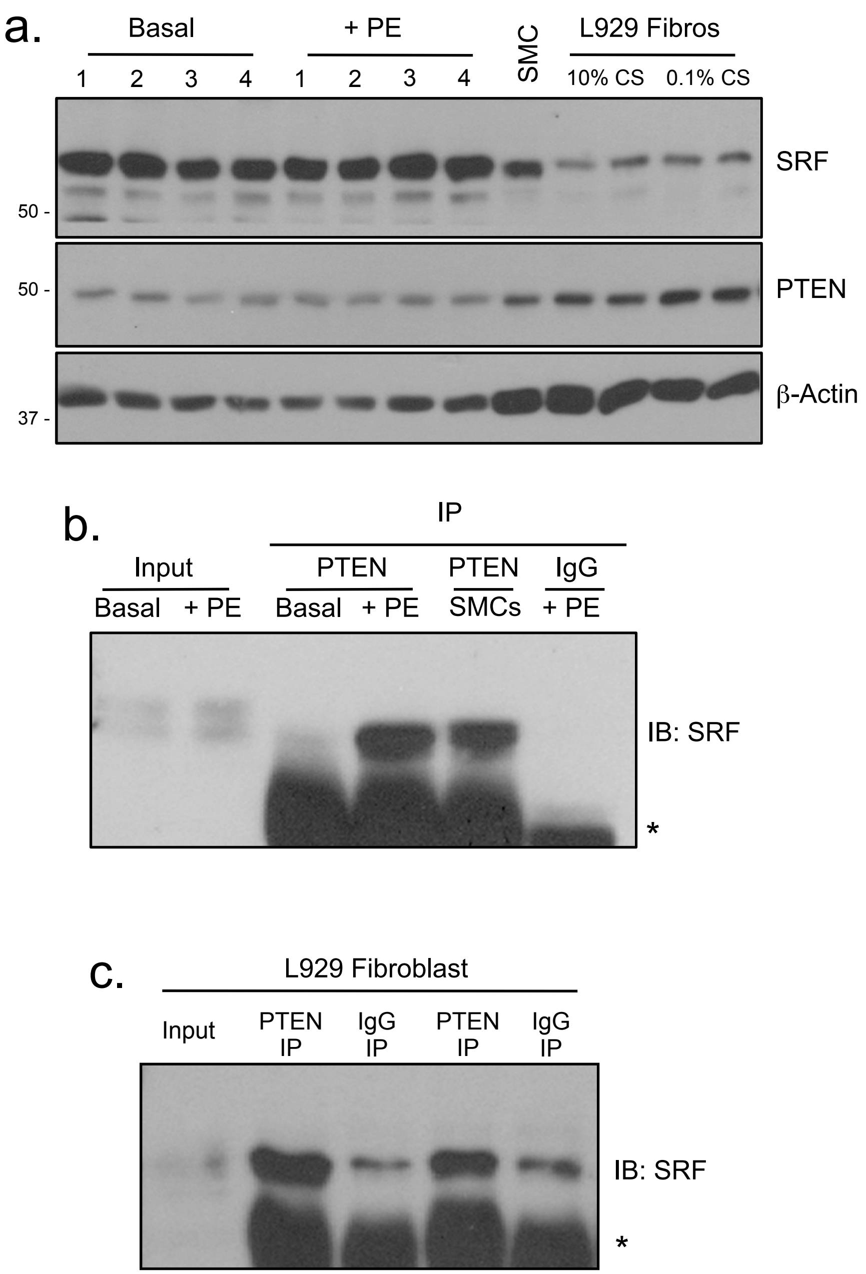

Supplementary Figure 4. PTEN and SRF interact in cardiomyocytes

(a). Neonatal rat ventricular myocytes (NRVMs) were cultured for 48 hrs under basal conditions or in the presence of 10 μm phenylephrine (PE). SMCs were serum restricted for 48 hrs. L929 fibroblasts were cultured for 48 hrs in 10% CS or 0.1% CS. Whole cells lysates were analyzed by Western blot for PTEN and SRF levels; β-actin was used as a loading control. Shown are four separate NRVM isolations per condition and two separate L929 isolations per condition. **(b).** PTEN was immunoprecipitated (IP) from WCL of serum restricted (Basal) NRVMs, NRVMs treated with 10 μm phenylephrine (+PE) for 48hrs, or SMCs serum restricted for 48 hrs. 10% of input NRVM WCL and co-immunoprecipitating SRF were detected by western blot. A non-specific IgG was used for IP on PE-treated NRVM as a negative control. *= heavy chain IgG. **(c).** PTEN was immunoprecipitated (IP) from WCL of L929 fibroblasts. 10% of input WCL and co-immunoprecipitating SRF was detected by immunoblotting. A non-specific IgG was used for IPs as a negative control. Shown are two independent IPs. Shown for each panel are representative images of N≥3 independent experiments. Molecular weight markers were cropped out for final SRF blots; please see Supplementary Figure 8.

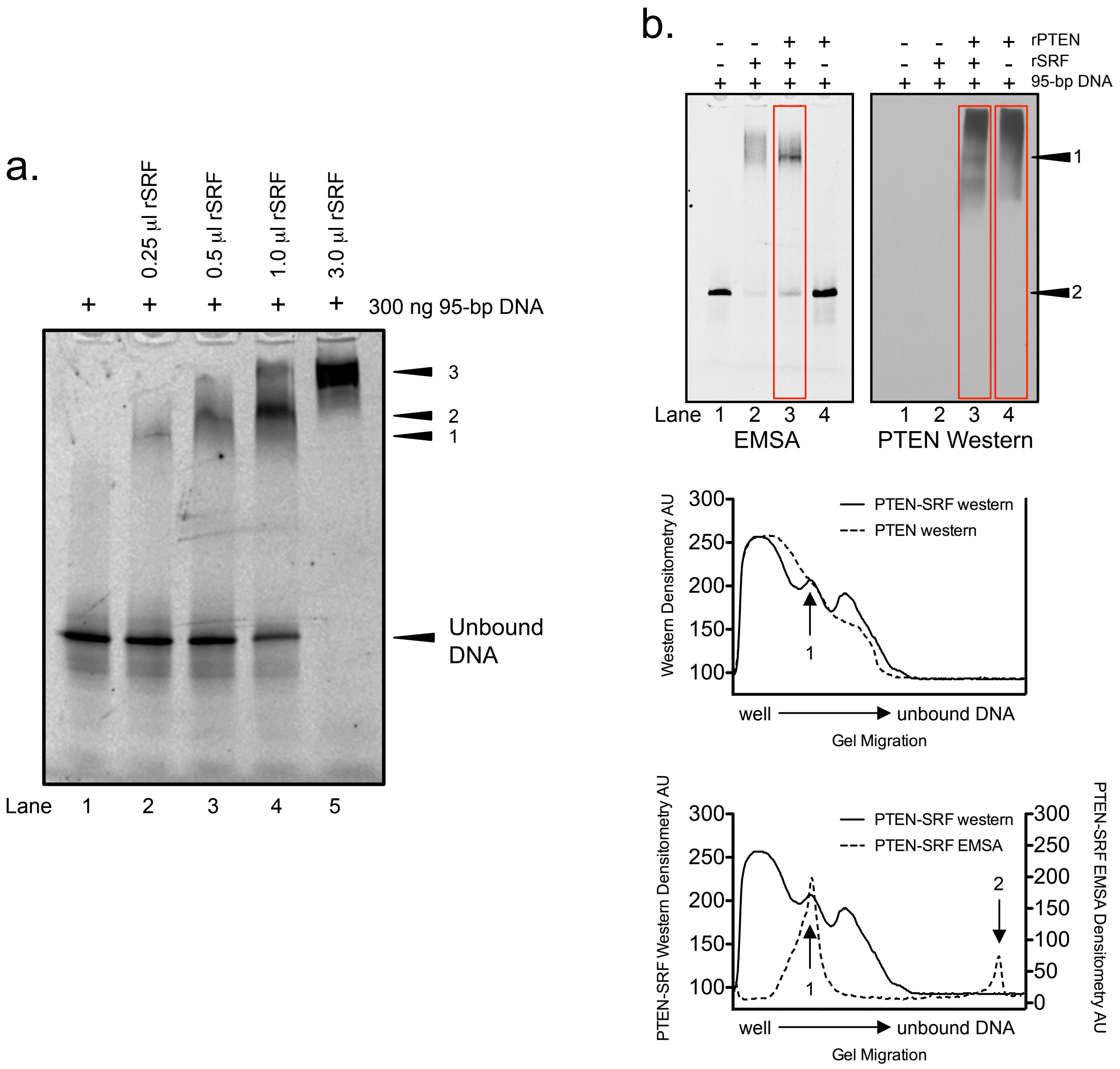

Supplementary Figure 5. PTEN interacts with SRF on CArG boxes of SM genes

(a). EMSA with 95-bp DNA fragment. DNA with increasing amounts of rSRF (lanes 2-5). DNA-SRF complexes are labeled bands "1-3". **(b).** EMSA with 95-bp DNA fragment. DNA plus rSRF alone (lane 2), DNA plus rSRF and rPTEN (lane 3), and DNA plus rPTEN alone (lane 4). EMSA was fluorescently imaged (upper left panel) then transferred to nitrocellulose and analyzed by western blotting for PTEN (upper right panel). Upper graph – overlay of densitometry scans of lanes 3 and 4 from the western blot. Lower graph – overlay of densitometry scans of lane 3 EMSA and lane 3 western blot. Red boxes indicate areas scanned for densitometry; "1" indicates PTEN western band/densitometry peak, lane 3 and SRF-PTEN-DNA complex band/densitometry peak on EMSA, lane 3; "2" indicates unbound DNA band/densitometry peak on EMSA, lane 3.

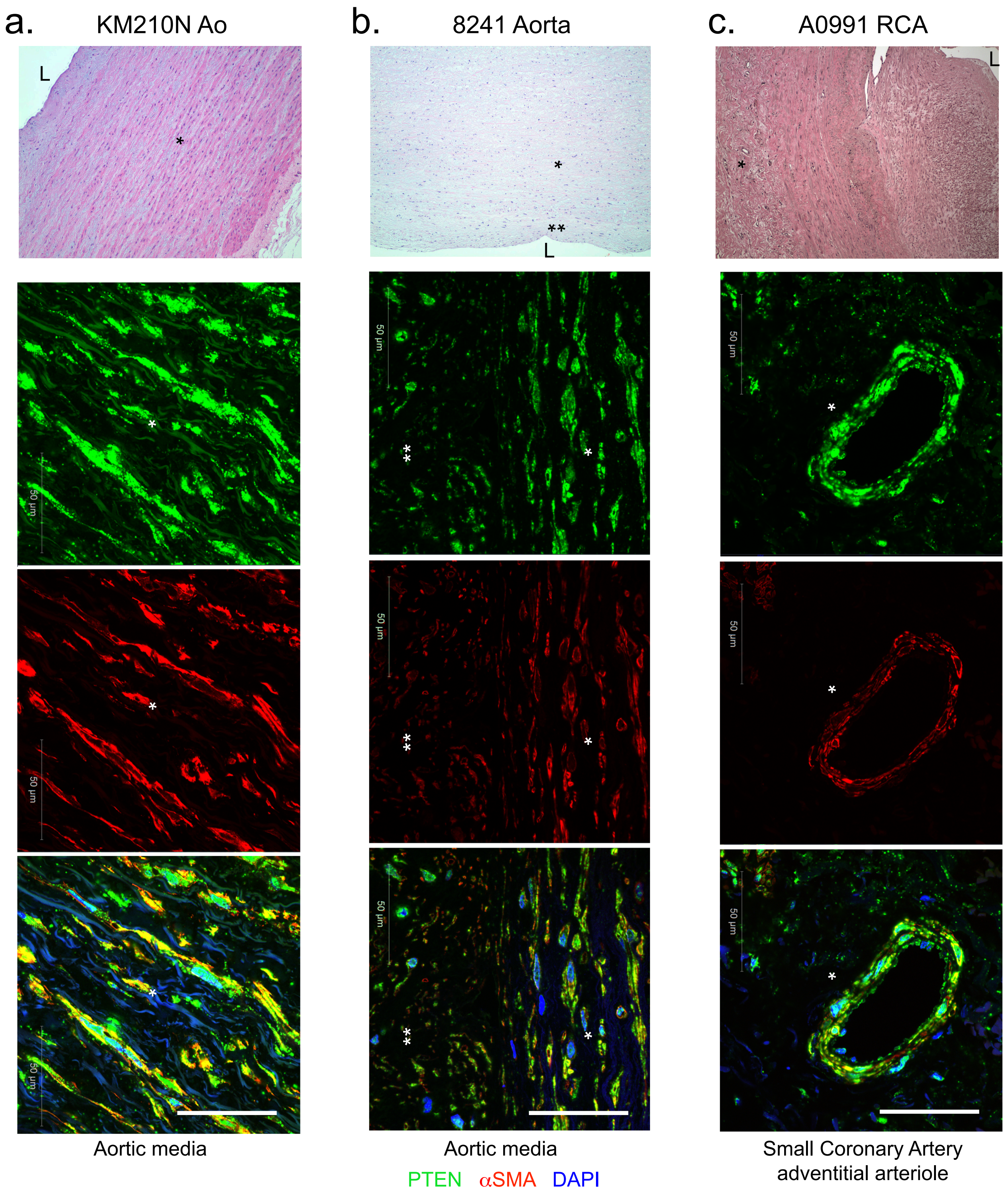

Supplementary Figure 6. Cytoplasmic and nuclear PTEN expression in medial SMCs of human atherosclerotic coronary arteries

Double immunofluorescent staining for PTEN (green) and αSMA (red) was conducted on arterial tissues from human patients; n=6 individual vessels/patients. For panels a-c, top panels are lower magnification H&E images of each vessel; * and ** represent the region of vessel shown in the fluorescent images. **(a-c).** Cytoplasmic and nuclear PTEN expression and co-localization of PTEN and αSMA in medial SMCs of normal aortic media (a&b) and a small adventitial artery (c). Scale bars = 50 μm.

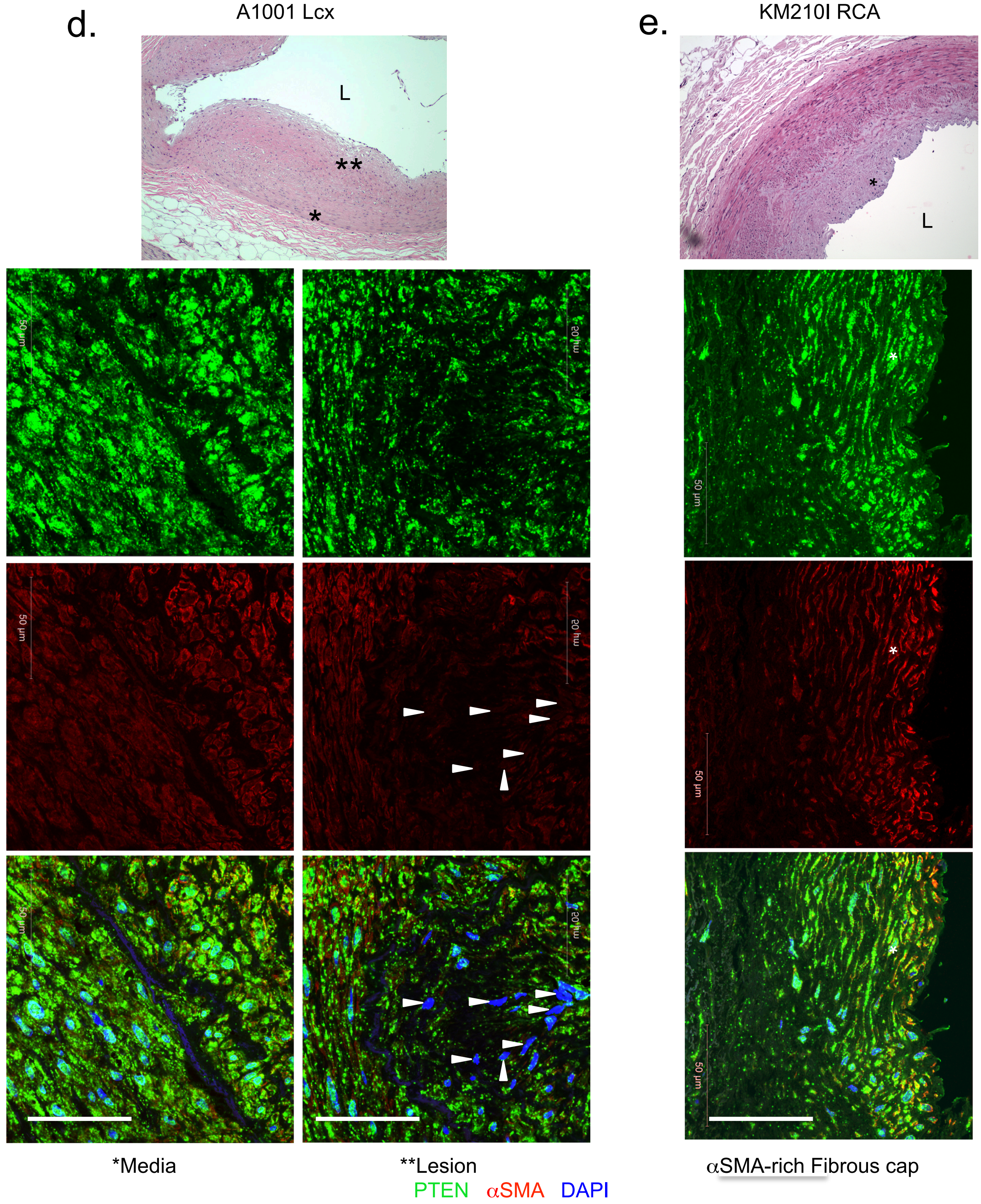

Supplementary Figure 6. Loss of nuclear PTEN expression in intimal SMCs of human atherosclerotic coronary arteries. Double immunofluorescent staining for PTEN (green) and αSMA (red) was conducted on arterial tissues from human patients; n=6 individual vessels/patients. For panels d&e, top panels are lower magnification H&E images of each vessel; * and ** represent the region of vessel shown in the fluorescent images. **(d).** Left circumflex artery with moderate atherosclerotic plaque demonstrating uniform cytoplasmic and nuclear PTEN staining in arterial medial SMCs (left panels), but loss of nuclear PTEN associated with loss of αSMA expression in intimal SMCs in plaque regions (arrowheads). **(e).** Right coronary artery with moderate atherosclerotic plaque demonstrating cytoplasmic and nuclear PTEN expression and co-localization of PTEN and αSMA in fibrous cap SMCs. Scale bars = 50 μm.

f.

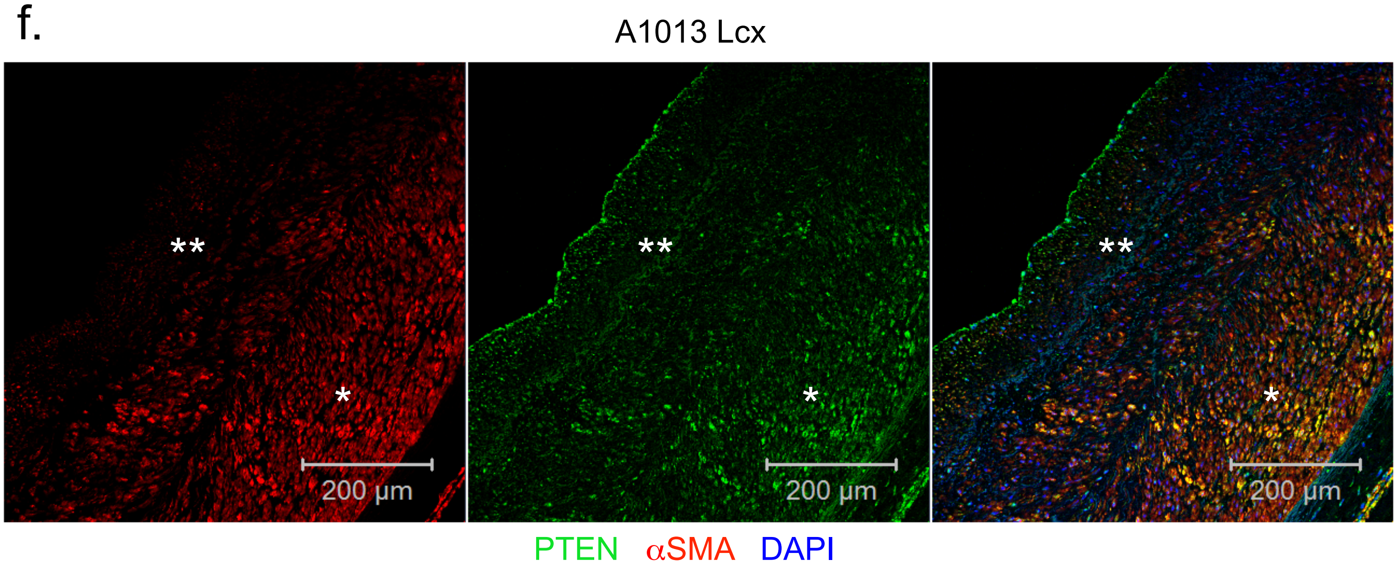

Supplementary Figure 6. Decreased PTEN expression in intimal SMCs of human atherosclerotic coronary arteries Double immunofluorescent staining for PTEN (green) and αSMA (red) was conducted on arterial tissues from human patients; n=6 individual vessels/patients. * and ** represent the region of vessel shown in the fluorescent images. **(f).** Lower power image of a left circumflex artery with moderate atherosclerotic plaque demonstrating decreased PTEN expression within plaque tissue that is associated with decreased aSMA expression. Tissue identification numbers and vessel description from de-identified specimens are listed above the images. Scale bars = 200 μm.

a. 1005131 Aorta

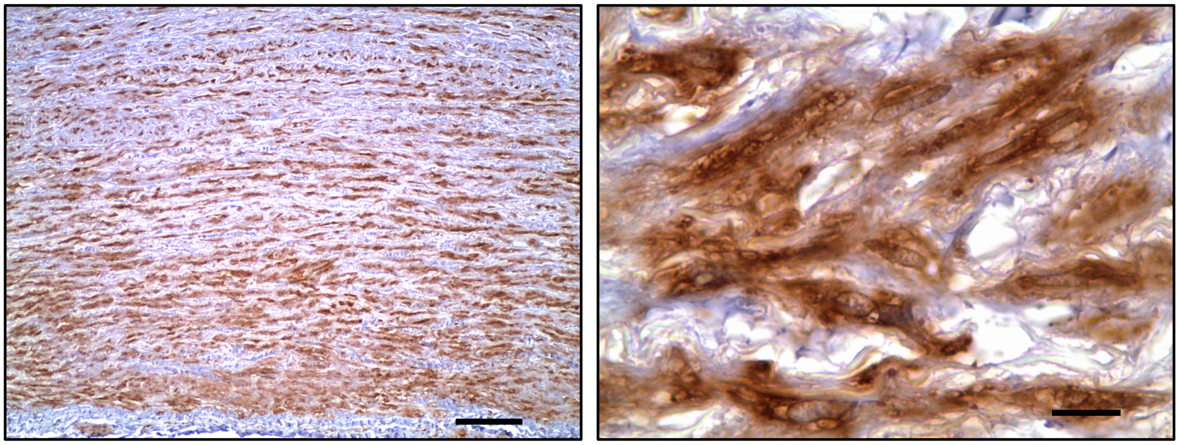

b. 105281 LAD2

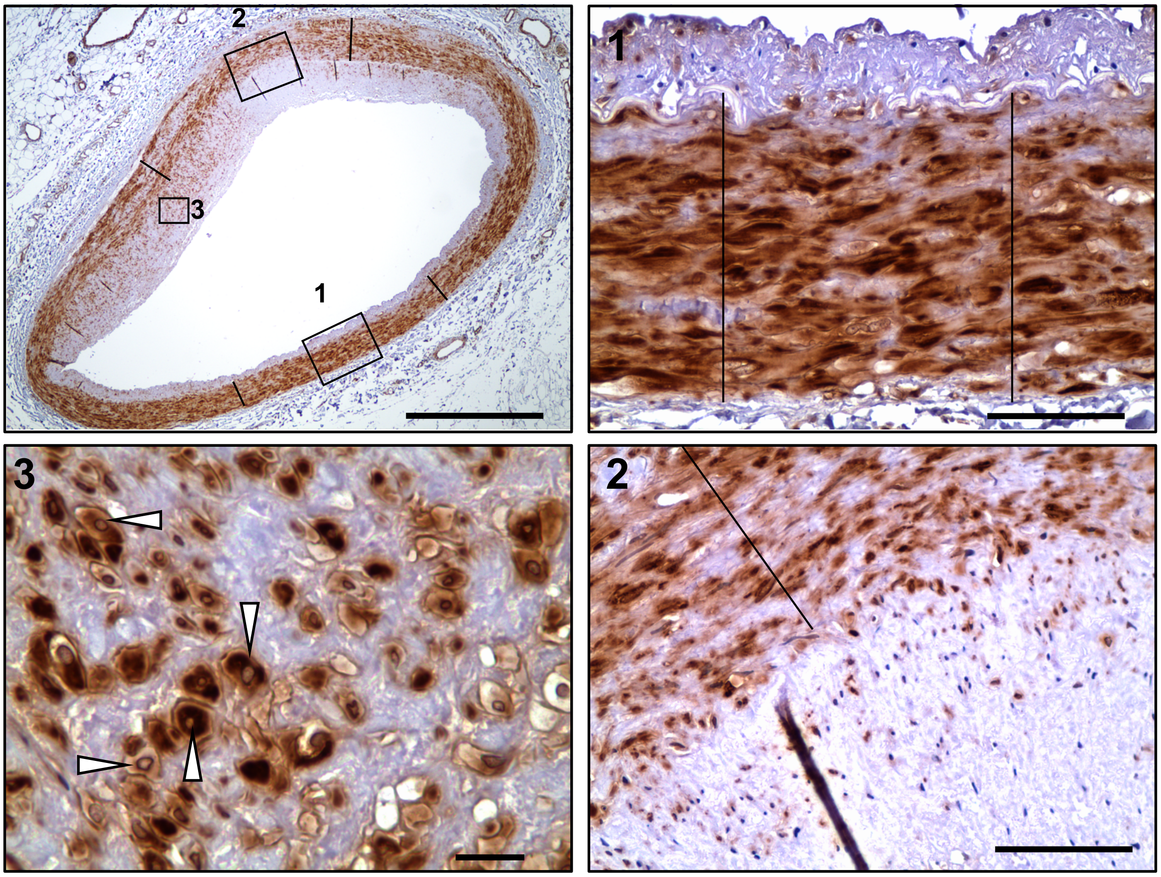

Supplementary Figure 7. PTEN expression in human normal and atherosclerotic arteries Immunohistochemical staining for PTEN expression was conducted on arterial tissues from human patients (brown reaction color); n=5 individual vessels/patients. **(a).** Normal aortic media showing uniform PTEN expression by medial SMCs. Left image shows low magnification; right image shows high magnification. **(b).** Left anterior descending coronary artery with mild intimal hyperplasia. Upper left panel: low magnification. Higher magnification of boxed, numbered regions shown in additional panels demonstrating uniform cytoplasmic and nuclear PTEN staining in medial SMCs (box 1), reduced or loss of PTEN expression in intimal cells (box 2), and PTEN nuclear exclusion in intimal cells (box 3; arrowheads). Tissue identification numbers and vessel description from de-identified specimens are listed above the images. Scale bars (a) = 200 μm (left) and 20 μm (right). Scale bars (b) = 1000 μm (upper left), 20 μm (lower left), and 100 μm (upper and lower right).

C.

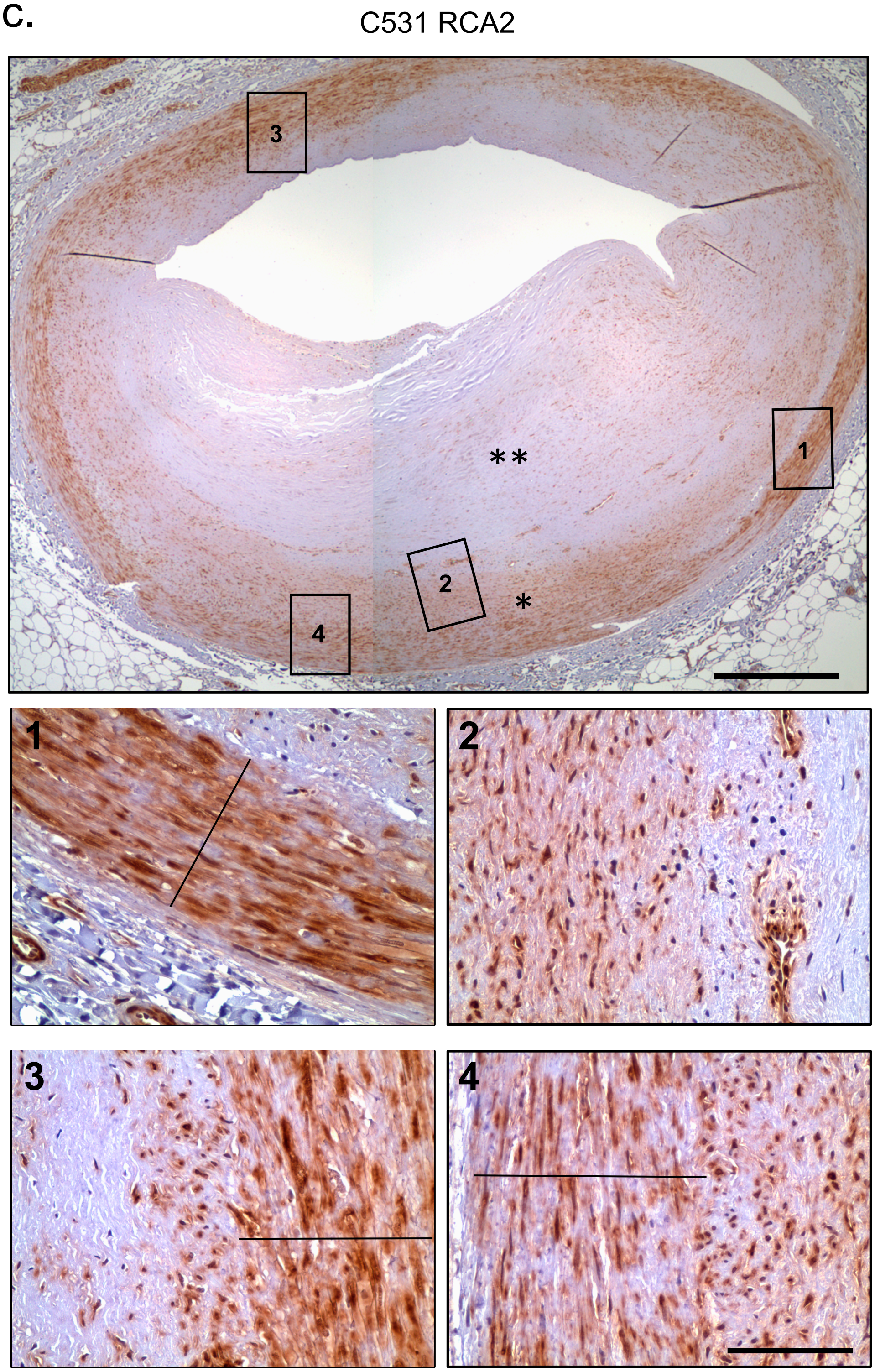

Supplementary Figure 7. Loss of PTEN expression in intimal SMCs of human atherosclerotic arteries Immunohistochemical staining for PTEN expression was conducted on arterial tissues from human patients (brown reaction color); n=5 individual vessels/patients. **(c).** Right coronary artery with moderate atherosclerotic plaque demonstrating decreased PTEN expression within plaque tissue. This vessel is the same vessel used for immunofluorescent staining shown in Figure 8b; * and ** represent arterial regions shown in Figure 8b. Upper panel: low magnification. Higher magnification of boxed, numbered regions shown in bottom panels. Tissue identification numbers and vessel description from de-identified specimens are listed above the images. Scale bar upper image = 1000 μm. Scale bars lower images (1-4) = 100 μm.

d.

C531 RCA1

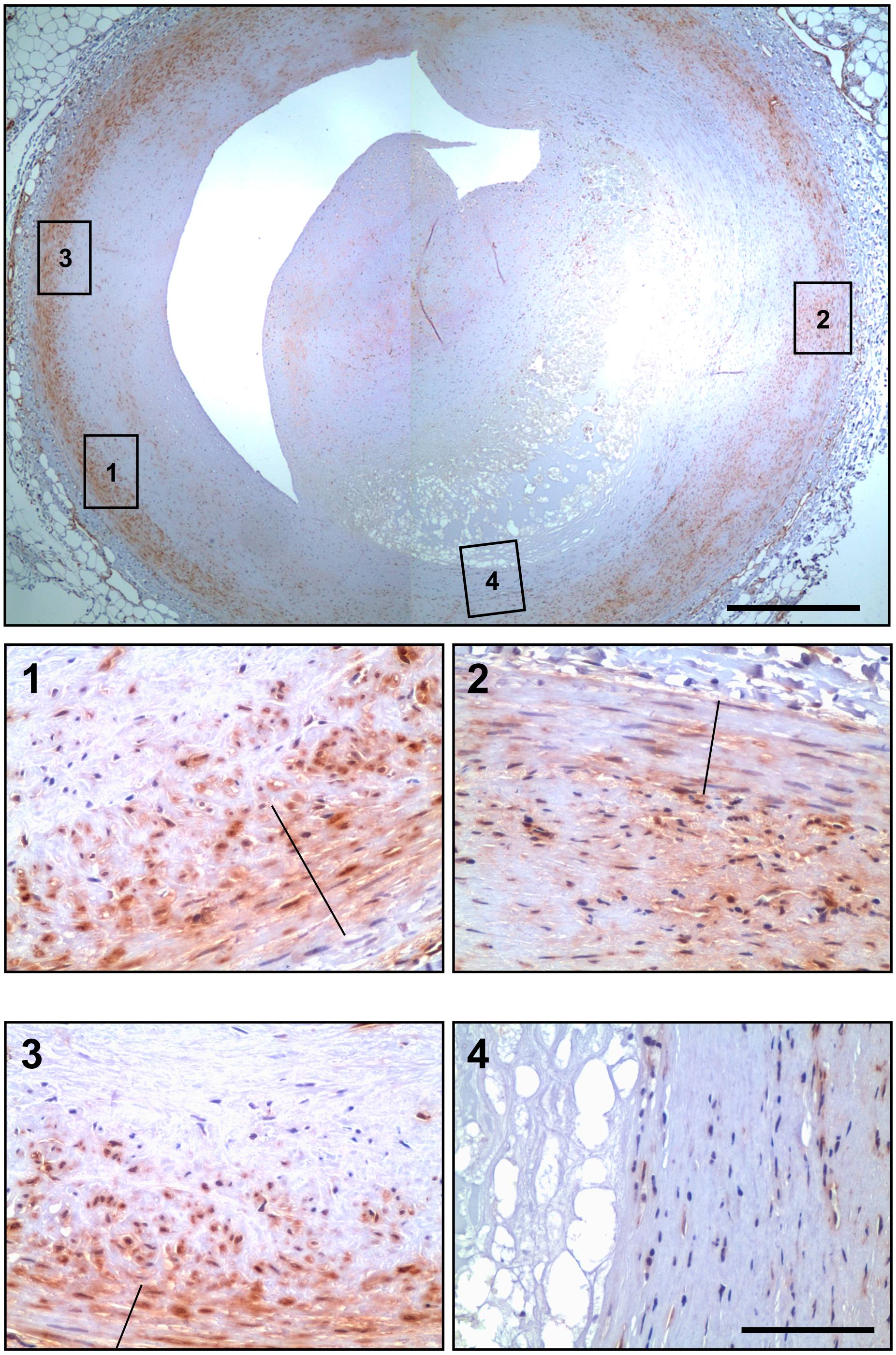

Supplementary Figure 7. Loss of PTEN expression in intimal SMCs of human atherosclerotic arteries Immunohistochemical staining for PTEN expression was conducted on arterial tissues from human patients (brown reaction color); n=5 individual vessels/patients. **(d).** Right coronary artery with large atherosclerotic plaque and intraplaque blood demonstrating overall decreased PTEN expression. Upper panel: low magnification. Higher magnification of boxed, numbered regions shown in bottom panels. Tissue identification numbers and vessel description from de-identified specimens are listed above the images. Scale bar upper image = 1000 μm. Scale bars lower images (1-4) = 100 μm.

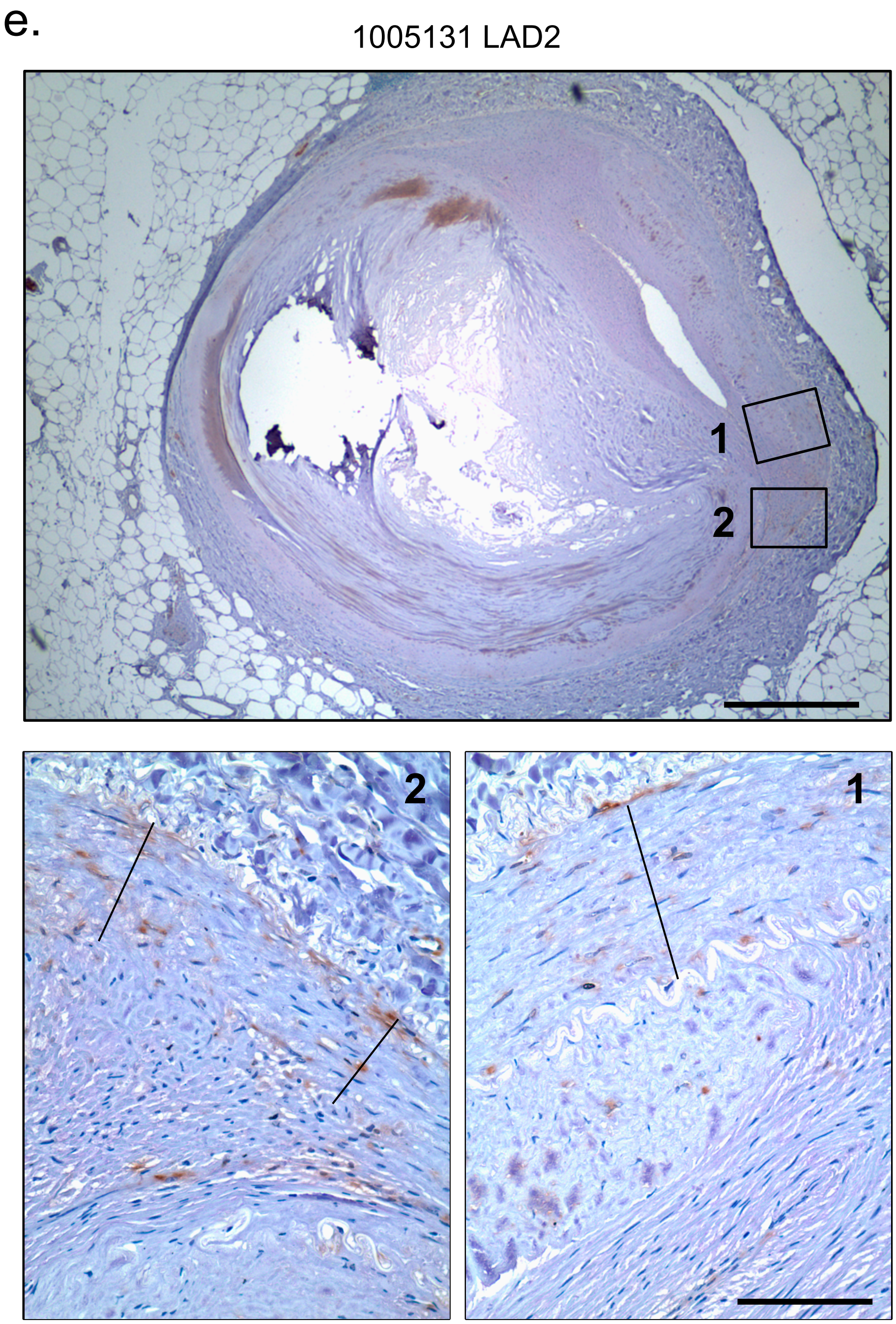

Supplementary Figure 7. Loss of PTEN expression in intimal SMCs of human atherosclerotic arteries Immunohistochemical staining for PTEN expression was conducted on arterial tissues from human patients (brown reaction color); n=5 individual vessels/patients. **(e).** Right coronary artery with large complex and ruptured plaque. Upper panel: low magnification. Higher magnification of boxed, numbered regions shown in bottom panels. Tissue identification numbers and vessel description from de-identified specimens are listed above the images. Scale bar upper image = 1000 μm. Scale bars lower images = 100 μm.

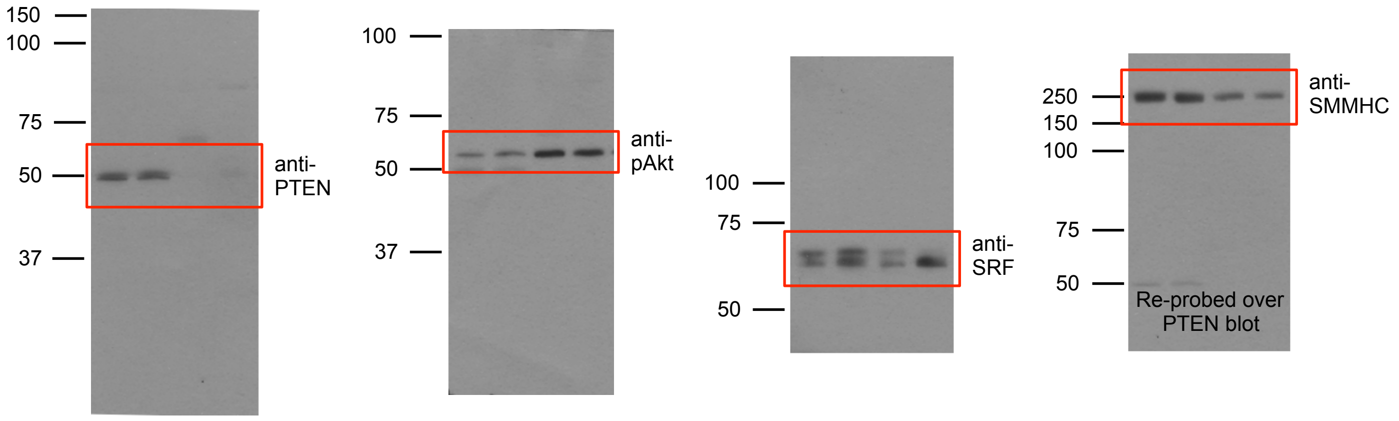

Figure 1C

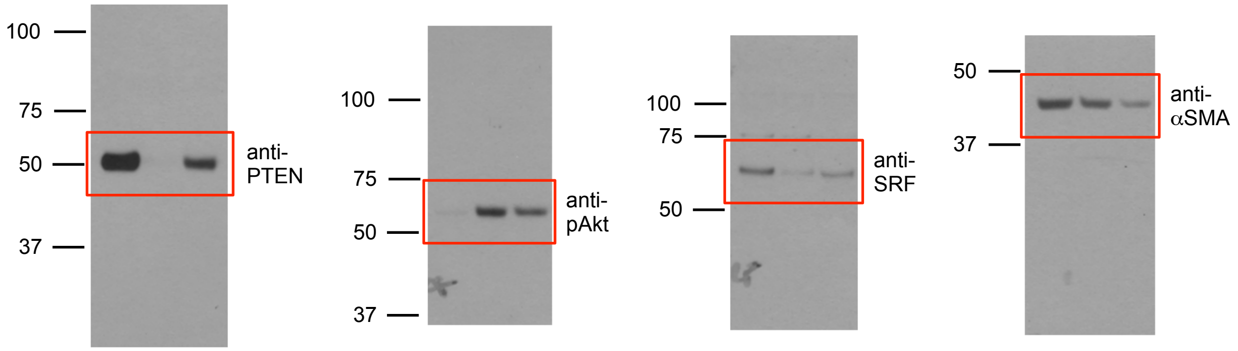

Figure 1D

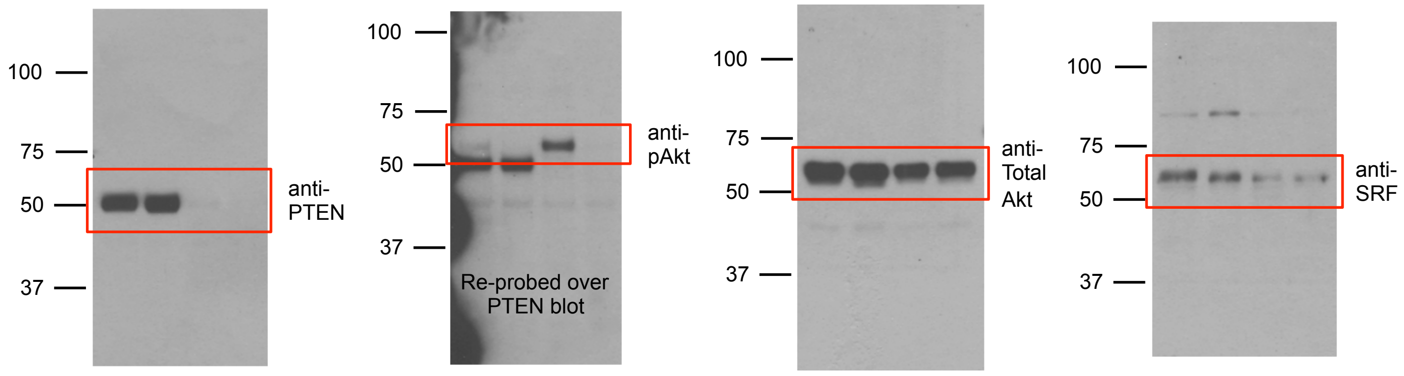

Figure 1F

Supplementary Figure 8. Uncropped Western Blots

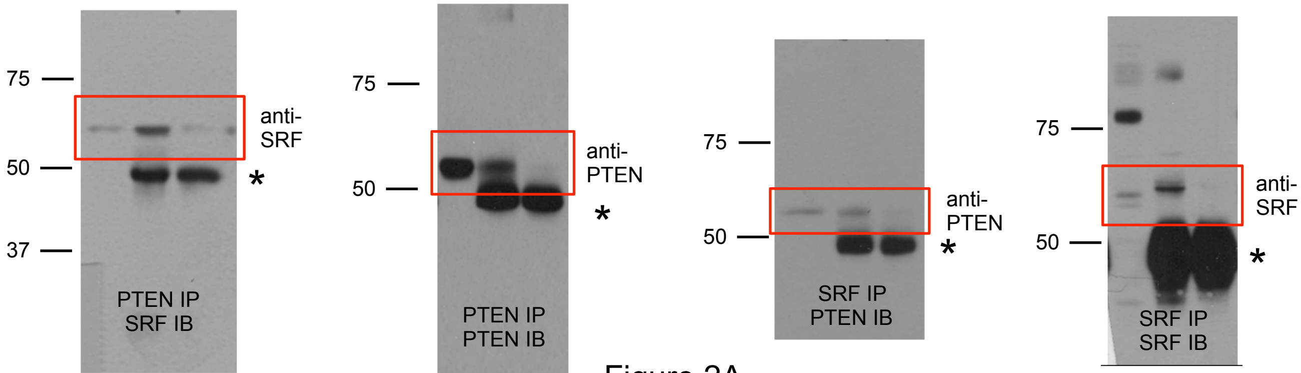

Figure 2A

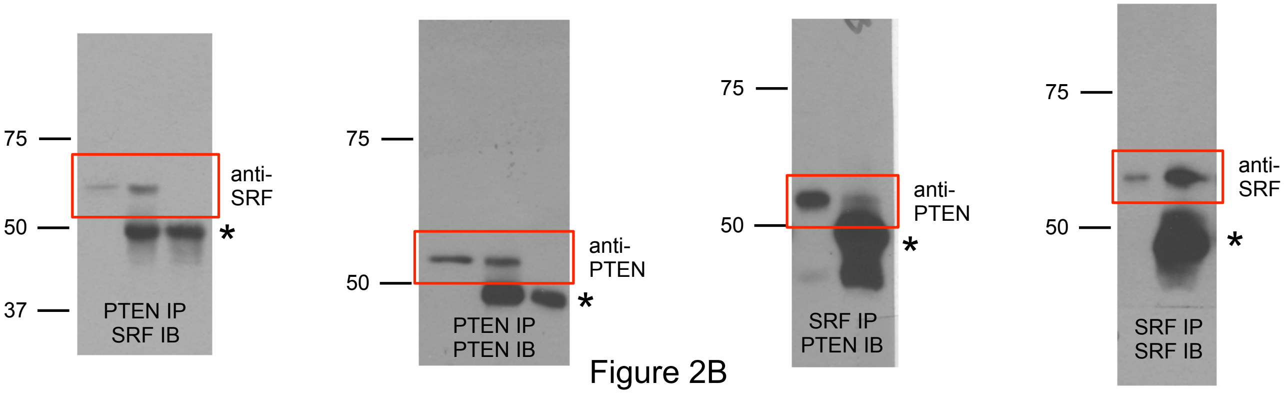

Figure 2B

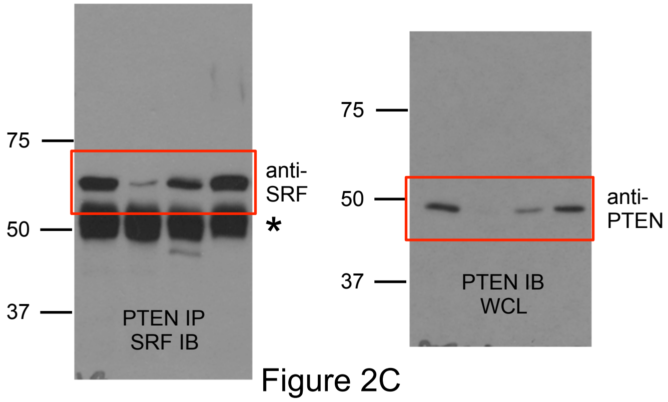

Figure 2C

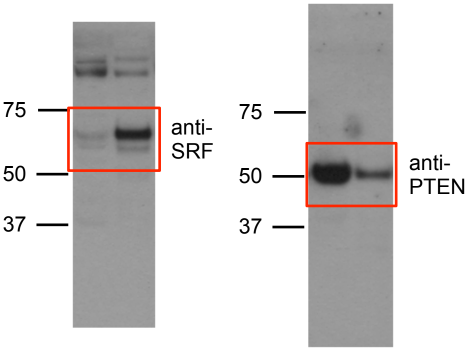

Figure 2D

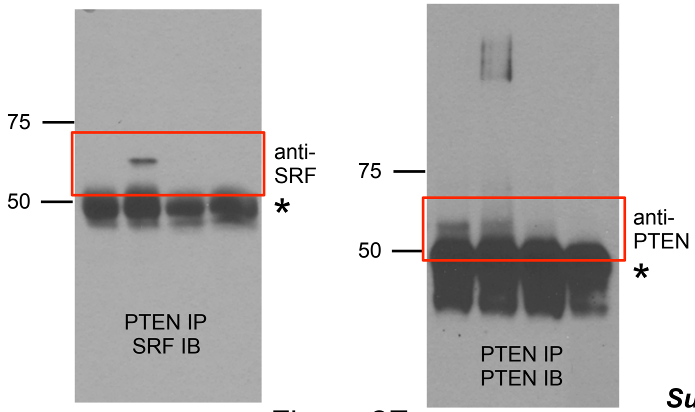

Figure 2E

Supplementary Figure 8. Uncropped Western Blots

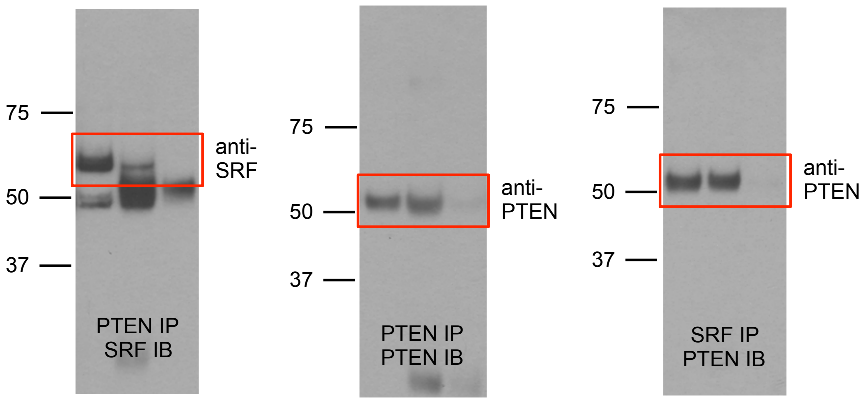

Figure 2F

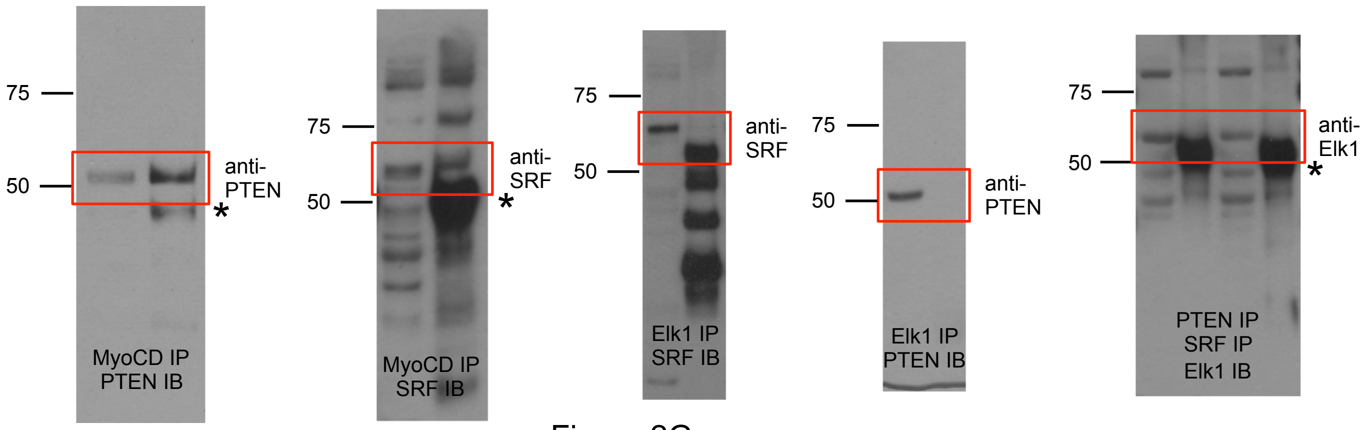

Figure 2G

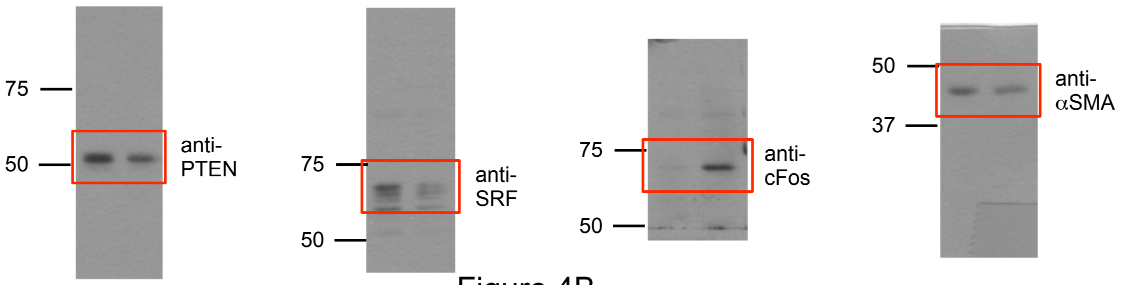

Figure 4B

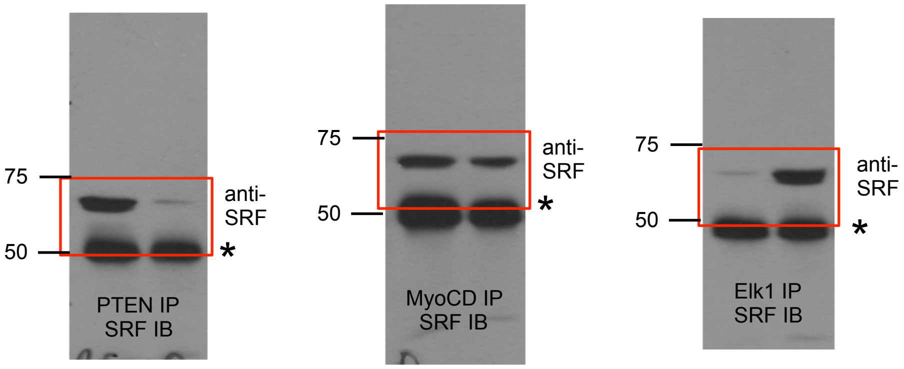

Figure 4C

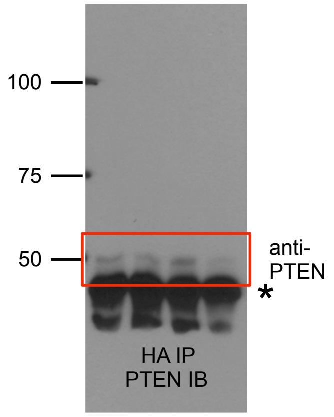

Figure 4E

Supplementary Figure 8. Uncropped Western Blots

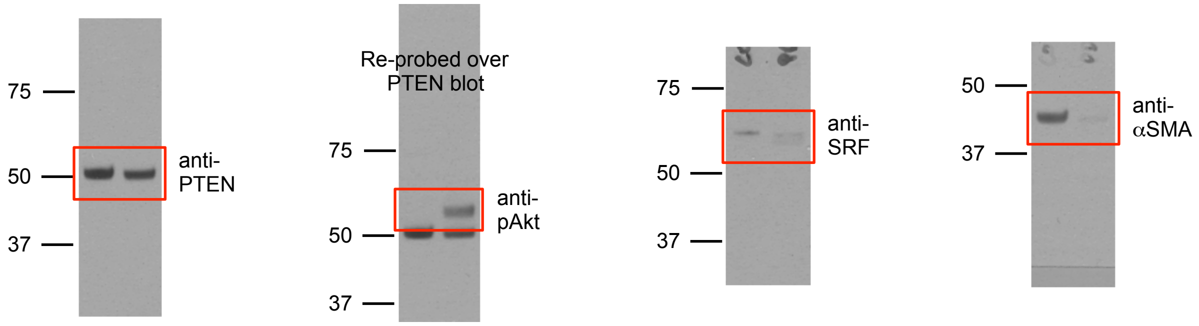

Figure 5A

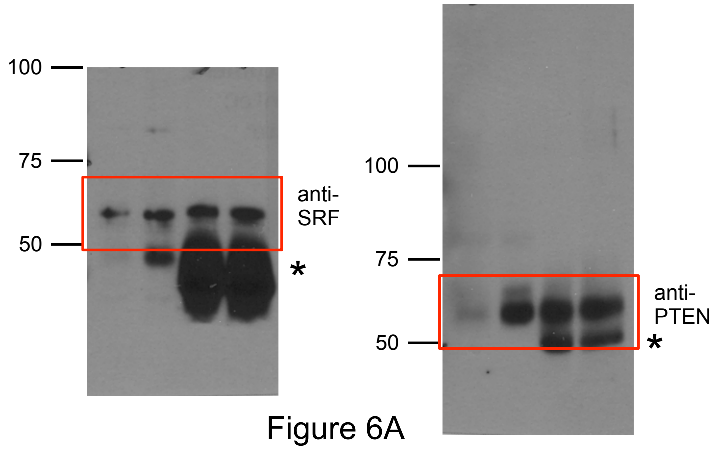

Figure 6A

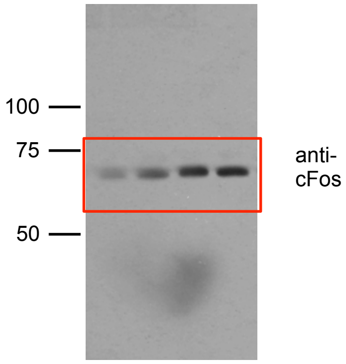

Figure 6I

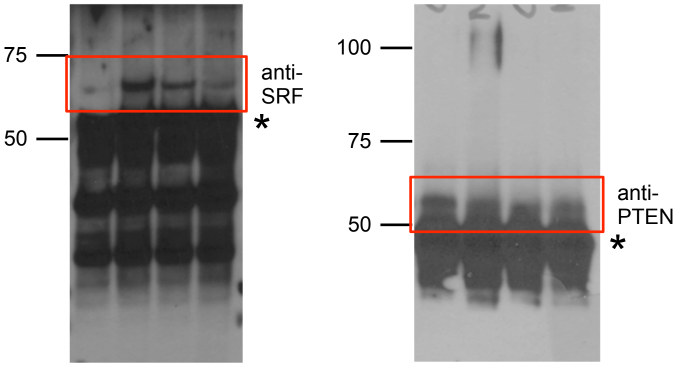

Figure 7B

Supplementary Figure 8. Uncropped Western Blots

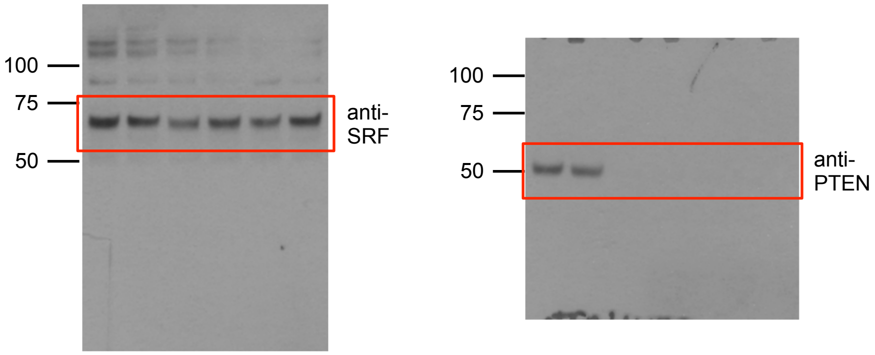

Supplementary Figure 1A

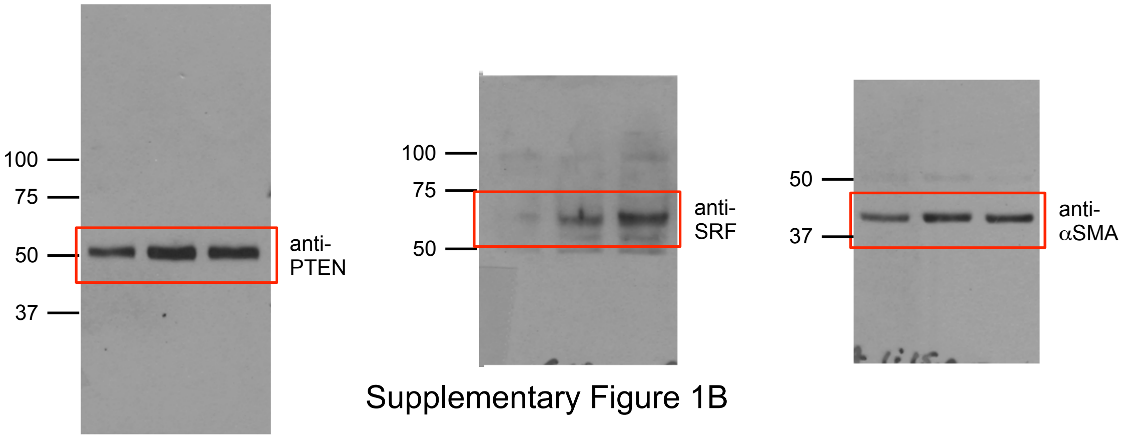

Supplementary Figure 1B

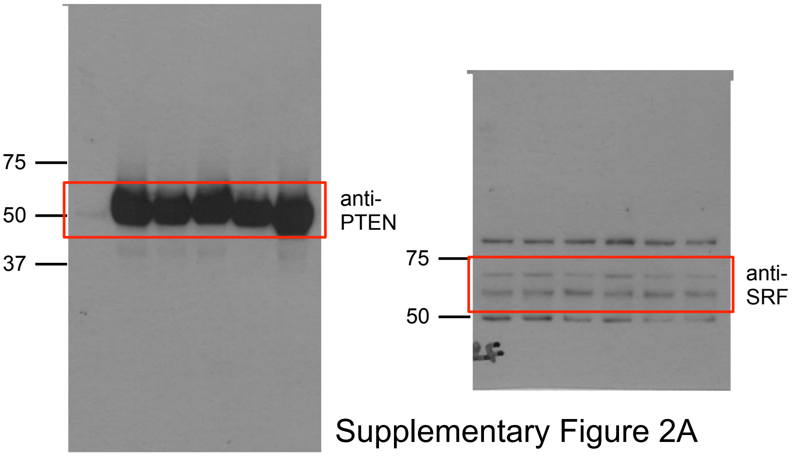

Supplementary Figure 2A

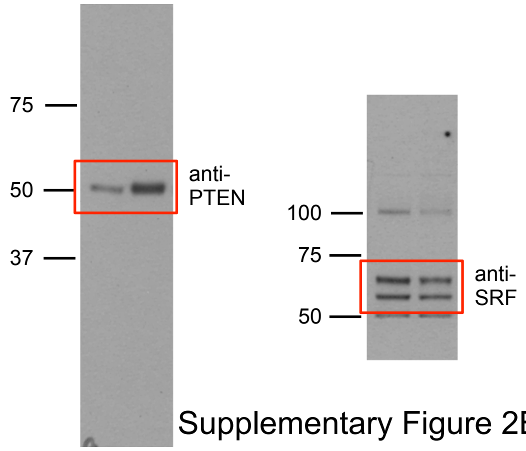

Supplementary Figure 2B

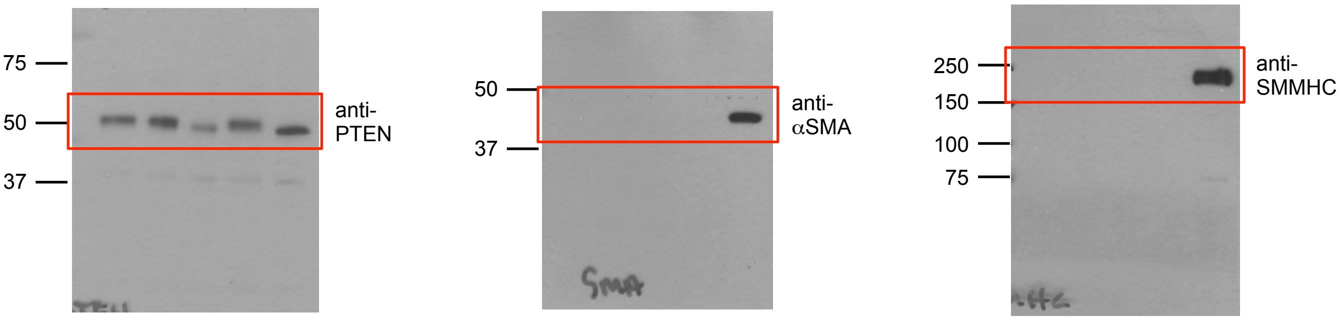

Supplementary Figure 2C

Supplementary Figure 8. Uncropped Western Blots

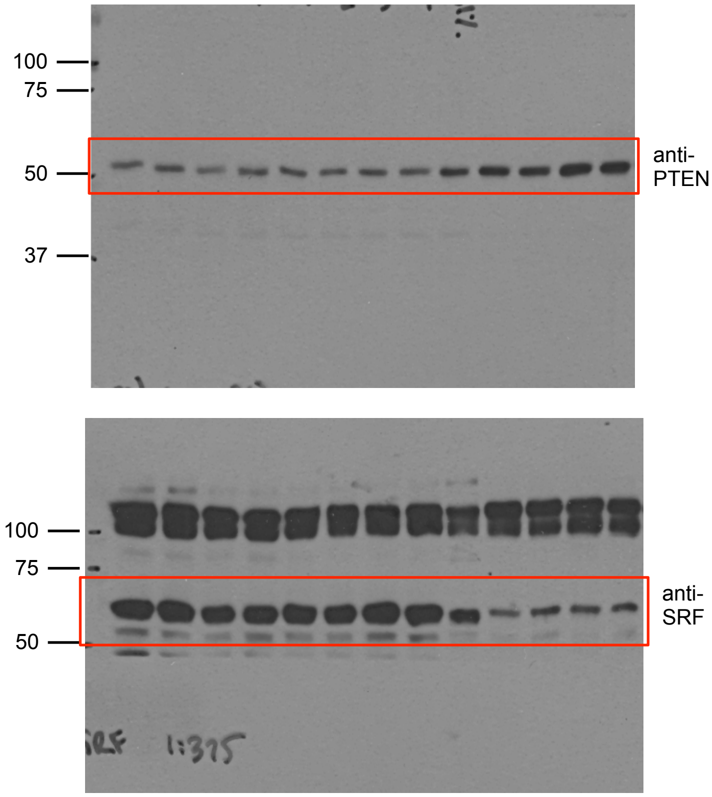

Supplementary Figure 4A

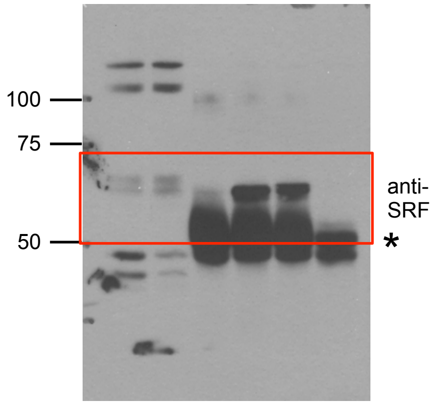

Supplementary Figure 4B

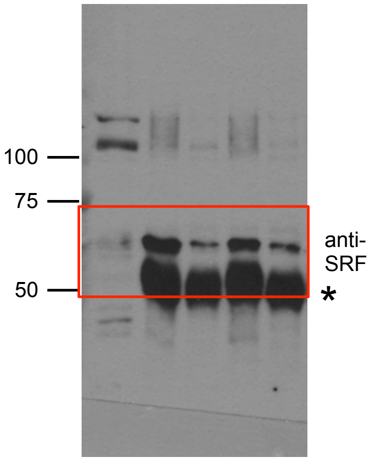

Supplementary Figure 4C

Supplementary Figure 8. Uncropped Western Blots
